# Antibody Repertoire Analysis of Hepatitis C Virus Infections Identifies Immune Signatures Associated With Spontaneous Clearance

**DOI:** 10.3389/fimmu.2018.03004

**Published:** 2018-12-21

**Authors:** Sivan Eliyahu, Oz Sharabi, Shiri Elmedvi, Reut Timor, Ateret Davidovich, Francois Vigneault, Chris Clouser, Ronen Hope, Assy Nimer, Marius Braun, Yaacov Y. Weiss, Pazit Polak, Gur Yaari, Meital Gal-Tanamy

**Affiliations:** ^1^Molecular Virology Lab, The Azrieli Faculty of Medicine, Bar-Ilan University, Safed, Israel; ^2^Bioengineering, Faculty of Engineering, Bar-Ilan University, Ramat-Gan, Israel; ^3^AbVitro, Inc., Boston, MA, United States; ^4^Internal Medicine Department A, Western Galilee Medical Center, Naharyia and Faculty of Medicine in the Galilee, Bar-Ilan University, Safed, Israel; ^5^Liver Institute, Rabin Medical Center, Sackler School of Medicine, Tel-Aviv University, Tel Aviv-Yafo, Israel

**Keywords:** hepatitis C virus, antibody repertoire, neutralizing antibodies, infectious disease, immune signature

## Abstract

Hepatitis C virus (HCV) is a major public health concern, with over 70 million people infected worldwide, who are at risk for developing life-threatening liver disease. No vaccine is available, and immunity against the virus is not well-understood. Following the acute stage, HCV usually causes chronic infections. However, ~30% of infected individuals spontaneously clear the virus. Therefore, using HCV as a model for comparing immune responses between spontaneous clearer (SC) and chronically infected (CI) individuals may empower the identification of mechanisms governing viral infection outcomes. Here, we provide the first in-depth analysis of adaptive immune receptor repertoires in individuals with current or past HCV infection. We demonstrate that SC individuals, in contrast to CI patients, develop clusters of antibodies with distinct properties. These antibodies' characteristics were used in a machine learning framework to accurately predict infection outcome. Using combinatorial antibody phage display library technology, we identified HCV-specific antibody sequences. By integrating these data with the repertoire analysis, we constructed two antibodies characterized by high neutralization breadth, which are associated with clearance. This study provides insight into the nature of effective immune response against HCV and demonstrates an innovative approach for constructing antibodies correlating with successful infection clearance. It may have clinical implications for prognosis of the future status of infection, and the design of effective immunotherapies and a vaccine for HCV.

## Introduction

HCV infection can lead to hepatitis, cirrhosis, liver failure, and hepatocellular carcinoma (HCC); it is the leading cause of liver transplantation (1). HCC is the fifth most common cancer, and the third leading cause of cancer-related death worldwide. Unfortunately, its prevalence in the US and Western Europe is increasing (1). No vaccine is currently available for HCV, and immunity against the virus is not well-understood. Cure rates are expected to increase with the recent approval of Direct-Acting Antiviral Drugs (DAAs). Yet, despite this progress, many challenges remain, such as limited implementation, efficacy, and protection from reinfection (2). Thus, global eradication of HCV by implementing DAAs is currently not a feasible goal (3–6). Since vaccination is considered the most effective means of eradicating viral infections (5), a prophylactic HCV vaccine is an urgent, unmet medical need (3–6). However, critical gaps in understanding the correlates of protective HCV immunity have hindered the design of anti-HCV vaccines and novel immunotherapeutics (3–6).

Unlike HIV-infections, which are not spontaneously cleared, 20–40% of HCV-infected individuals experience spontaneous recovery (7). A multitude of evidence suggests that induction of an efficient HCV-specific natural immunity can control the infection. Therefore, using HCV as a model for comparing immune responses between spontaneous clearer (SC) and chronically infected (CI) individuals will enable the identification of unique mechanisms that govern human disease outcomes. Until recently, protection against persistent HCV infection was thought to be associated with a vigorous T-cell response (8). However, it is now widely accepted that neutralizing antibodies (nAbs) also play a key role in viral clearance (8–12). This point was strengthened by demonstrating that natural clearance correlates with the early development of nAbs (13), and with nAbs that exhibit distinct epitope specificity (14). Extensive characterization of monoclonal HCV-neutralizing antibodies (mnAbs), combined with crystal structures of the HCV envelope protein E2, which is the target of most HCV-nAbs, has provided valuable information regarding the E2 antigenic landscape (15–19). However, since most HCV mnAbs characterized to date were generated from CI patients (12, 20, 21), the nature and epitope specificities of mnAbs in SC individuals remain to be elucidated. Recent studies have demonstrated that the early appearance of broadly neutralizing antibodies (bnAbs) is associated with spontaneous clearance (13). Interestingly, bnAbs also protect against HCV infection in animal models (22–24). Very recently, the first panels of bnAbs isolated from SC infections have been developed (25, 26). The panel reported by Bailey et al. displayed a low number of somatic mutations compared with the well-characterized nAbs from chronic patients exhibiting higher neutralization breadth, but were similar to nAbs from chronic infections in terms of clonality and epitope specificities (26). It remains unknown whether and how the immune response of SC individuals is distinct from that of CI patients.

New emerging technologies empowering high-throughput direct screening for specific antibodies have provided deep insights into the immunogens that elicit broad antibody responses (27, 28). In the case of HIV, such technologies led to the generation of broadly neutralizing monoclonal antibodies with significantly higher potency, breadth, and novel epitope specificities [reviewed in (29)]. These novel revolutionary methods of studying immune responses can offer important insights into the nature of immune responses to infections. The antibody repertoire of an individual stores information about current and past threats that the body has encountered, and thus has the potential to shed light on screening antibodies and vaccine design (27, 30). Comparing the features of antibody repertoires between distinct patient populations may provide information that can be correlated with clinically relevant outcomes (31, 32). Indeed, recent studies have found common antibody sequences in unrelated individuals following Dengue (33), influenza (34), and HIV (35) infections, as well as autoimmune diseases such as celiac (36) and pemphigus vulgaris (37). In chronic lymphocytic leukemia, 30% of patients carry highly similar antibodies (38). Here we utilized high-resolution technologies to identify unique antibodies that stratify between CI and SC HCV infection outcomes. We also used antibody repertoires in combination with phage display to construct HCV-specific broadly nAbs associated with HCV infection clearance.

## Materials and Methods

### Cell Lines

Huh-7.5 cells (a generous gift from Charles Rice, Rockefeller University) and Huh7/FT3-7 cells (a generous gift from Stanley M. Lemon, University of North Carolina at Chapel Hill) are human hepatoma cell lines that are highly permissive for infection and replication of cell culture infectious HCV (HCVcc) (39). Cells were cultured in Dulbecco's modified Eagle's medium (DMEM) containing high glucose; 10% fetal bovine serum (FBS); 1% L-glutamine; 1% penicillin streptomycin; and 1% non-essential amino acid. The cells were incubated in a humidified incubator at 37°C containing 5% CO_2_. The irradiated 3T3-msCD40L feeder cells that express CD40L were obtained from the National Institutes of Health (NIH) and cultured as previously described (40).

### Virus

Virus stocks from HJ3–5 chimeric virus [a generous gift from Stanley M. Lemon, University of North Carolina at Chapel Hill (39)] and the other chimeric viruses containing E2 envelope protein from genotypes 1-7: HJ3-5/1a, H77C/1a, j6/2b, s52/3a, ED43/4a, sa13/5a, HK6A/6a, QC69/7a [a generous gift from Jens Bukh (41)], were produced in Huh7/FT3-7 cells and viral titers were determined by FFU assay in Huh-7.5 cells, as described previously (39).

### Antibodies

A panel of HCV mnAbs CBH-4B, CBH-4D, HC-1, HC-11, CBH-7, HC84.22, HC84.26, HC33.1, and HC33.4 that are representative E2 antigenic domain A-E antibodies, and a control non-specific antibody R04 (12, 20, 21) were kindly provided by Steven Foung, Stanford Univ., Stanford, California.

### Sample-Collection

All blood samples were collected from the Liver Institute at Belinson and the Galilee Medical Center, Israel. In total, we obtained blood samples from 80 individuals; of these, 18 were individuals that spontaneously cleared HCV infection, 52 were with persistent chronic HCV infections, and 10 were from healthy controls. Subjects were defined as spontaneously cleared HCV if anti-HCV antibodies are detectable, with undetectable HCV RNA assessed by the Taqman reverse-transcription polymerase chain reaction (RT-PCR) quantitative assays. HCV chronic infections were defined as viremia if there were detectable viral loads for more than 1 year. Both cohorts were not treated with any anti-viral treatment. All blood samples were collected using protocols approved by the Institutional Review Boards and were in accordance with the ethical standards of the Helsinki Declaration. Sample data are summarized in Supplementary Table 1. For the isolation of peripheral blood mononuclear cells (PBMCs), 30–50 ml of whole blood from each donor was separated on Ficoll-Paque gradient (Lymphoprep™) according to the manufacturer's instructions.

### Expression and Purification of the E2 Glycoprotein

The H77 genotype 1a E2 sequence (GenBank accession no. AF009606), spanning residues 384–661 (not containing the transmembrane domain), was amplified by PCR using HCV plasmid pHJ3-5 (39) and primers pSHOOTER-sec-E2-1a-SE and pSHOOTER-sec-E2-1a-As (primers are listed in Supplementary Table 2). The PCR product was digested with NotI and NcoI and cloned into plasmid pCMV-SEC-MBP (a generous gift from Itai Benhar, Tel-Aviv University, Tel-Aviv, Israel) containing signal peptide for secretion, His and Myc tags, and fused to maltose-binding protein (MBP) for higher expression and stabilization. The resulting plasmid was termed pCMV-SEC-MBP-E2-384-661-1a-His-Myc.

For production of E2 protein, 293T cells were transfected with 12 μg pCMV-SEC-MBP-E2-384-661-1a-His-Myc expression plasmid by PEI transfection reagent. At 72 h post transfection, medium containing the secreted protein was collected from cells for protein purification. The E2 protein was purified using Ni-NTA agarose beads (Qiagen) according to the manufacturer's instructions. Purified E2 glycoprotein was stored at −20°C. E2 glycoprotein-containing fractions were analyzed on SDS 10% polyacrylamide gels.

### Construction of an Immune anti-HCV Antibody Phage Display Library

We constructed a phage display antibody library from a source of pooled PBMCs obtained from 10 SC patients. For library construction, we designed a degenerative primer set by using the IMGT database (IMGT®, the international ImMunoGeneTics information system® http://www.imgt.org (founder and director: Marie-Paule Lefranc, Montpellier, France) (42) (primers are listed in Supplementary Table 2). The phage antibody library was produced using a protocol as previously described (43). In brief, total RNA was extracted from 10^7^ PBMCs using the RNeasy mini kit (Qiagen). cDNA was produced from mRNA by reverse transcription using the AccuScript Hi-Fi cDNA Synthesis Kit (Agilent). Heavy and light chain variable domains were amplified from the RT-PCR cDNA product by PCR using the primer sets we have designed. The heavy variable domains were amplified using the primer sets Hu-VH1-6-NcoI-BACK and Hu-JH1-6-FORF and the light variable domain was amplified using primer sets Hu-VK1-6-BACKF and Hu-JK1-5-NotI-FORF (for amplifying Kappa light chains) or Hu-VL1-10-BACKF and Hu-JL1-7-NotI-FORF (for amplifying Lambda light chains). For the combinatorial assembly of the heavy and light chain variable domains into complete single-chain variable fragments (scFv), the fragments were mixed according to their natural frequencies, and PCR was performed using the assembly primer (forward) and the primers set Hu-JK1-5-NotI-FORF for Kappa scFv or the primers set Hu-JL1-7-NotI-FORF for Lambda scFv (reverse) (primers are listed in Supplementary Table 2). The amplified scFvs were cloned into the phagemid vector pCC16 (43). The ligated DNA was used for electroporation into electrocompetent XL-1 cells (Agilent Technologies) under the following conditions: 2.5 kV, 200 Ω, 25 μF. In total, we conducted 75 electroporations that yielded a total library size of 6^*^10^7^ individual clones. To test the diversity of the libraries, we amplify the scFv genes from 30 colonies from the library by PCR. The PCR products were digested by BstNI (NEB). The digested samples were separated on 2.5% agarose gel. A diverse running pattern indicates sequence diversity. Rescue of the library using helper phage and preparation of library stocks was performed essentially as described (43).

### Biopanning and Isolation of Monoclonal Anti-E2 Phages

To enrich E2-specific phages, five cycles of biopanning were performed for the SC library essentially as described (43). In brief, phages were first rescued from the library. Then, the first cycle of enrichment was performed by coating the wells with E2 glycoprotein, and then 10^11^ phages were added to the wells. Non-specific phages were washed by PBST and then specific phages were eluted with 100 mM triethylamine. For neutralization, 1 M Tris∙Cl pH 7.4 was added. Eluted phages were used for the next cycle of biopanning. Phages were pooled from the 4th and 5th biopanning cycles. Next, 96 colonies were picked from each cycle and rescued essentially as described (43). Their specificity to E2 was screened by ELISA, as described below.

### Expression and Purification of Full-Length Antibodies

To produce full-length IgGs, the heavy and light chains from scFvs were cloned into pMAZ-IgH and pMAZ-IgL vectors (a generous gift from Itai Benhar, Tel-Aviv University) that contain the constant regions of IgG1and a signal peptide for secretion (44). The variable heavy chain region was recovered by PCR from pCC16 vector, which carries the selected scFv using primers TAB-RI and CBD-As (Supplementary Table 2). Alternatively, the variable Heavy chain region sequences identified and selected by bioinformatic analysis were custom-synthesized (IDT, Israel). The variable Kappa and Lambda chain regions were recovered by PCR from pCC16 vector, which carries the selected scFv using primers TAB-RI and CBD-As (Supplementary Table 2). PCR products were digested with *BssH*II and *Nhe*I for heavy chains, *BssH*II and *Bsiw*I for the light Kappa chain, and *BssH*II and *Avr*II for the light Lambda chain, and cloned into the appropriate vectors.

For antibody production, 293T cells were transfected with pMAZ-IgH expressing the Heavy chain and with pMAZ-IgL expressing the Light chain. At 72 h post transfection, medium was collected from the cells and antibodies were purified using Protein A Sepharose CL-4B beads (GE healthcare) according to the manufacturer's instructions. Purified antibodies were stored at −20°C. Fractions containing antibodies were analyzed on SDS 15% polyacrylamide gels.

### ELISA

#### For Detecting Specific Antibodies in Patients' Sera

Each well of the ELISA plate was coated with 0.5 μg of rE2 diluted in 100 μl of coating buffer and the plates were incubated at 4°C overnight. The plates were washed twice with PBST and blocked with 3% skim milk in PBS for 1 h at 37°C. Next, the plates were washed twice with PBST and serum (diluted 1:1,000) from different patients were added to the wells, followed by 1 h incubation at RT. The plates were washed three times with PBST and goat α human HRP-conjugated antibody diluted 1:10,000 was added to each well, followed by 1 h incubation at RT. Then, 100 μl of Tetramethylbenzidine (TMB) was added to each well and following incubation of 5–10 min, the reaction was stopped by adding 50 μl of H_2_SO_4_ 0.5 M to each well. The signal was detected at a wavelength of 450 nm by a plate reader.

#### For Detecting Binding Phages

ELISA was performed as previously described (43). First, 96-well ELISA plates were coated with 5 μg of rE2 or negative control protein (BSA). Plates were incubated overnight, then washed × 3 with PBS, and blocking buffer was added to the plates for 2 h at 37°C. Next, individual rescued phages were added from the master plate. Plates were incubated at RT for 1 h and washed ×3 with PBS. Next, 1:5,000 HRP conjugated to α M13 antibody was added. Then, 100 μl of TMB was added and following an incubation of 30 min, the reaction was stopped by adding 50 μl of H_2_SO_4_ 0.5 M to each well. The signal was detected at a wavelength of 410 nm by a plate reader. Specific phages were picked by detection of positive signal for rE2 compared with BSA.

#### For Determining Antibodies' Specificity

For detecting antibodies binding to rE2, ELISA plates were coated with 5 μg of rE2. The plate was incubated and blocking buffer was added. Then, antibodies were added in concentration of 16 μg/ml and incubated for 1 h at RT. HRP-conjugated Goat α Human was added at 1:10,000 dilution and the plate was incubated for 1 h at RT. TMB was added and following an incubation of 5–10 min, 50 μl of H_2_SO_4_ 0.5 M was added to each well. The signal was detected at a wavelength of 450 nm by a plate reader.

### Focus-Forming Unit (FFU) Reduction Neutralization Assay

Neutralization assays were carried out essentially as we described previously (45). Huh7.5 cells were seeded on an eight-chamber slide and incubated overnight at 37°C. The next day, 5^*^10^11^ of each selected phage or different concentrations of purified IgGs were incubated for 1 h with 100 FFU of HCVcc HJ3-5 chimeric virus or viruses containing E2 from genotypes 1–7 [1a (H77/JFH1); 2b (J8/JFH1); 3a (S52/JFH1); 4a (ED43/JFH1); 5a (SA13/JFH1); 6a (HK6a/JFH1); 7a (QC69/JFH1)]. Next, phages/IgGs and virus mixtures were added to the wells. The slides were incubated for 24 h. Next, 200 μl of DMEM was added to each well and the slide was incubated for another 24 h. Then, the slides were washed twice with 200 μl PBS. The PBS was gently removed and 100 μl of Methanol:Acetone 1:1 was added to each well, followed by 10 min incubation at RT. Each well was washed twice with 200 μl PBS. Then 7.5% BSA in PBS was added with serum from a CI HCV patient at a dilution of 1:1,000, followed by 1 h of incubation at 37°C. Each well was washed twice with 200 μl PBS. Next, 100 μl of 7.5% BSA in PBS with fluorescently labeled goat anti-human antibody diluted 1:100 was added to each well, followed by 1 h of incubation at RT. Each well was washed 3 times with 200 μl PBS. Neutralization was measured by immunofluorescence microscopy, followed by manual counting of foci of infected cells. The percent neutralization was calculated as the percent reduction in FFU compared with virus incubated with an irrelevant control antibody.

### Isolation of HCV-Specific B Cells

We established a platform for the propagation and isolation of HCV-specific B cells. PBMCs from CI and SC patients were isolated and CD19^+^ B cells were separated by a FACS sorter. B cells were then plated on feeder irradiated 3T3-msCD40L cells that express CD40L, which induces proliferation, Ab class switching, and secretion (46). B cells were activated with 5 μg/ml rE2 protein and a combination of IL2 (10,000 U/ml) and IL21 (100 μg/ml) (47). The combination of CD40L feeder cells and the addition of cytokines IL2 and IL21 can successfully stimulate switched memory B cells to produce high concentrations of IgG to the supernatant.

Supplementary Figure 1 demonstrates the successful propagation of memory B cells following separation of CD19+ B cells from a healthy individual, that were grown on 3T3-msCD40L cells and stimulated with a pool of positive peptides and IL2 and IL-21. Evaluation of CFSE staining following 14 days of culture demonstrates CFSE fading, only under stimulated conditions. This indicates the proliferation of the activated culture (Supplementary Figure 1A). Moreover, in the activated culture, 23% of the population was memory B-cells that are positive for CD27+, compared with very low numbers of CD27+ cells in the non-activated culture (Supplementary Figure 1B). For evaluating the ability of B cells to differentiate and produce IgG, we measured the concentrations of IgG secreted to the culture medium 3 or 8 days following B-cell activation by ELISA. As shown in Supplementary Figure 1C, the activation induced IgG secretion, in a time and cell number-dependent manner.

For isolation of HCV-specific B-cells, B-cells from CI, and SC patients were isolated and stimulated as described above. The cultures were incubated for 14 days and then HCV-specific B cells were isolated. Activated B cells were incubated with rE2 and stained with CD19-PE, CD27-BV421, and tagged rE2 (anti-cMyc, alexa fluor 633). Viable CD19^+^, CD27^+^, and E2^+^ were isolated by FACS. These HCV-specific B cells were then grown for 1 week, as described above. Supernatants were collected at each step and used in the HCV-neutralization assays. The background was compared to healthy individuals, stained, and gated as the tested samples.

### Sequencing B-Cell Repertoires

#### Library Preparation

Total RNA was purified from 5^*^10^6^ PBMCs from each sample (using RNeasy Mini kit, Qiagen). RT-PCR was performed using an oligo dT primer. An adaptor sequence was added to the 5' end, which contains a universal priming site and a 17-nucleotide unique molecular identifier (48–51). Products were purified, followed by PCR using primers targeting the IgD, IgM, IgG, and IgA regions, and the universal adaptor. PCR products were then purified using AMPure XP beads. A second PCR was performed to add the Illumina P5 adaptor to the constant region end, and a sample-indexed P7 adaptor to the universal adaptor. Final products were purified, quantified with a TapeStation (Agilent Genomics), and pooled in equimolar proportions, followed by 2 × 300 paired-end sequencing with a 20% PhiX spike on the Illumina MiSeq platform according to the manufacturer's recommendations.

### Bioinformatic Analyses

Pre-processing of raw sequencing reads: Repertoire Sequencing TOolkit (pRESTO version 0.5.8) (52) was applied to the raw reads using the following steps: (a) Removal of low-quality reads (mean Phred quality score < 20). (b) Removal of reads where the primer could not be identified or had a poor alignment score (mismatch rate >0.1). (c) Identification of sets of sequences with identical molecular IDs (corresponding to the same mRNA molecule). These are collapsed into one consensus sequence per set, after removing sets with a mean mismatch rate >0.2. (d) Assembly of the two consensus paired-end reads into a complete antibody sequence. Then, V(D)J segments were assigned for each of the antibody sequences using IMGT/HighV-QUEST (53). This was followed by quality control and additional filtering: (a) Removal of non-functional sequences due to a stop codon or a reading frame shift between the V and the J gene. (b) Sequences with CDR3 length < 12 nucleotides. (c) Samples with an unusually abundant single V-J CDR3 length combination were excluded: samples CI4 and SC12 met this criterion, since they had a single sequence in >50% of the raw reads. (d) For mutation analysis sequences with read numbers (CONSCOUNT) lower than two were removed. (e) For IGHV gene usage we showed analysis for only functional genes that were in the 15 topmost frequent in at least one sample.

### Clustering of Related B-cell Sequences Across all Samples

Sequences were first grouped according to their V-gene, J-gene, and CDR3 length. For each group, the difference in amino acids between each pair of CDR3s was calculated by Hamming distance. Hierarchical clustering by a complete linkage method was applied and sequences were clustered by genetic distance, using a threshold of 0.15, i.e., the maximal dissimilarity between any two CDR3 sequences in a cluster never exceeded 15%. As an additional quality control step, sequence clusters for which >90% of sequences came from a single sample were removed.

### Comparing HCV-Specific B Cells and General Repertoires From SC and CI Clinical Groups by Amino Acid Conservation Levels

The frequency of each amino acid (AA) at each CDR3 position was calculated for each B-cell cluster. The sums of frequency squares were calculated for each clinical group. B-cell clusters containing CDR3 positions for which the sum of frequencies in SC was greater than the corresponding sum for CI by more than 0.5 were selected. Only clusters with sequences originating from more than one sample, and sequences with CONSCOUNT >1 were used.

### Prediction Model Based on the Patients' Repertoire

1. Sequences were grouped to clusters as described above.
2. The frequency of each cluster per sample was calculated.
3. A classification model was applied as follows:
  - The data set was randomly divided into 18 (~90%) and 2 samples (~10%) of training and test sets, respectively.
  - Feature selection was performed by a random forest model, choosing the most informative 18 features.
  - Logistic regression with an L2 regularization penalty was applied to these 18 remaining features, and the model was applied to the test set. The accuracy rate was measured.
  - The process was repeated 100 times; each time two different samples were taken as a test set.
  - Random predictions: to ensure that our results are not biased, clinical group labels were randomly shuffled. Then, steps a-d were applied to this permuted labels model.
4. A similar model was applied to T-cell repertoires, except that clusters of sequences were defined by identical CDR3 regions at the amino acid level.

### Data Availability

The antibody repertoires sequencing datasets for this study were deposited in the European Nucleotide Archive. The accession numbers are ERR2843386-ERR2843427.

## Results

**Figure 1:**
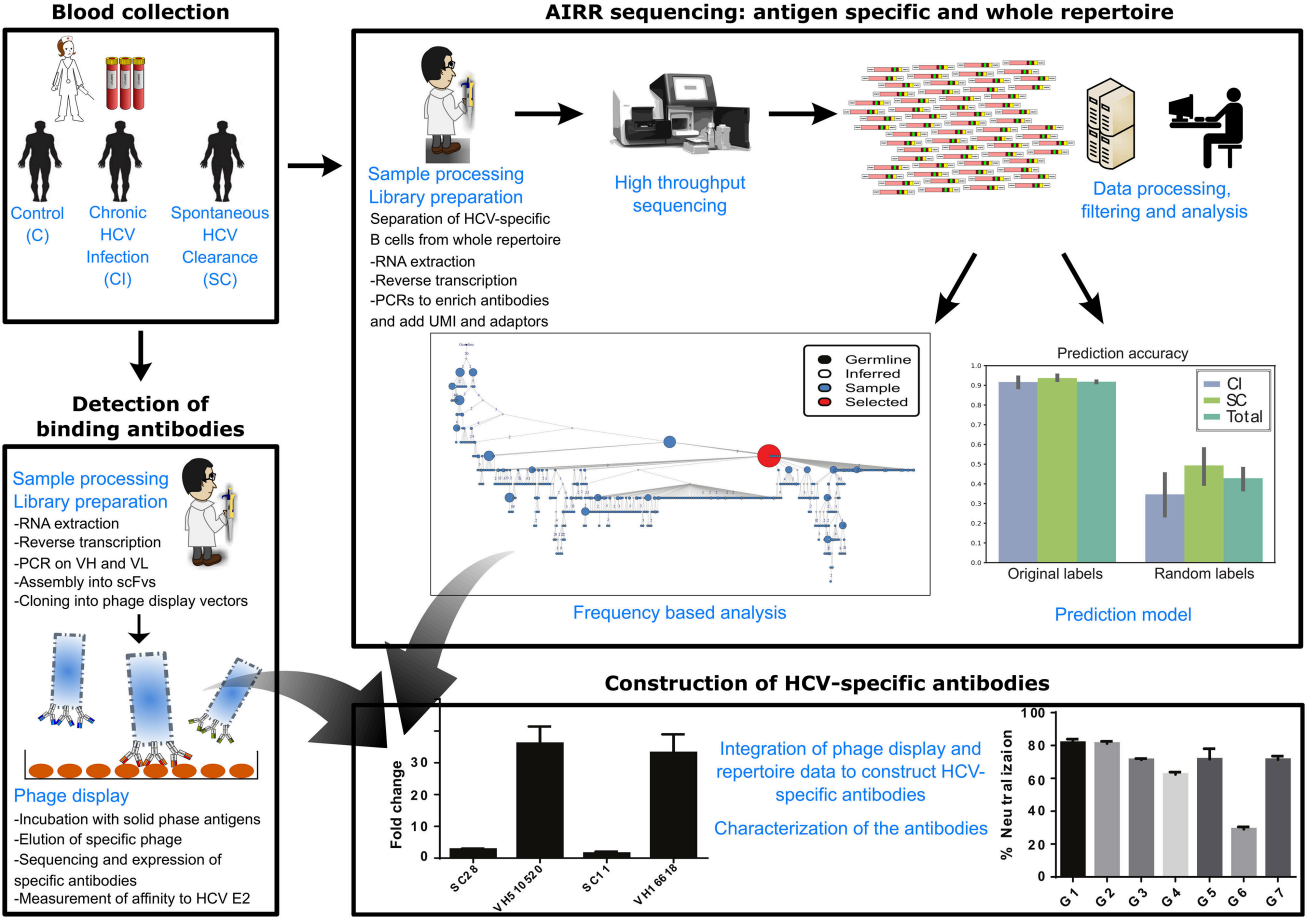
Scheme of workflow. The workflow included the following steps: collection of blood samples from SC, CI, and healthy individuals, sequencing of total B-cell repertoires, T-cell repertoires, and HCV-specific B-cell repertoires, analysis of repertoires and identification of antibody clusters and TCR sequences associated with viral clearance, construction of an antibody phage display library, isolation of a panel of HCV-binding antibody sequences that associate with cleared infections, and integration of all data to construct HCV-broadly neutralizing antibodies associated with clearance.

Our overall approach is summarized in Figure 1; it included a collection of blood samples from CI and SC HCV infections in addition to healthy controls, and a screen to identify samples containing high levels of HCV-nAbs. Selected samples were used for sequencing of total and HCV-specific antibody repertoires, as well as total T-cell receptor repertoires. This was followed by constructing monoclonal antibodies associated with infection clearance, based on phage display antibody library and repertoire data (Figure 1).

### Anti-HCV Antibodies in Resolved Infections Are Potent Neutralizers

We collected PBMCs and sera from 80 individuals. Of these, 18 were individuals that spontaneously cleared HCV infection, 52 were with persistent chronic HCV infection, and 10 were from healthy controls. To validate the presence of nAbs in sera from CI and SC HCV infections, we first screened these sera by ELISA for antibodies able to bind a recombinant HCV envelope protein E2 (rE2) that we have produced. Although high levels of anti-rE2 were detected in chronic HCV infections, very low levels were detected in resolved HCV infections (Supplementary Figure 2A). This is expected, since the ongoing infection in CI patients results in the generation of large numbers of anti-HCV antibodies from plasma cells, whereas in resolved individuals, anti-HCV antibodies are secreted from lower number of circulating HCV-specific long lived plasma cells or memory B-cells. Then, we screened these sera for HCV-neutralization by performing an HCVcc neutralization assay. Approximately a 2-fold drop in neutralization efficiency was observed in resolved infections (an average of 45%) compared with chronic infections (an average of 85%) (Supplementary Figure 2B).

To validate that we indeed measured HCV-specific immunity, we collected two CI samples before and after successful anti-viral therapy (SVR). The blood samples were collected between 6 months and 1 year after achieving SVR. Using these samples, we again tested binding to rE2 and HCV-neutralization. As expected, we observed a significant drop both in binding and in neutralizing HCV following treatment (Supplementary Figures 2C,D). Collectively, these results suggest that although the anti-HCV antibodies in resolved infections are at low levels, they are potent neutralizers. The samples that displayed high neutralization efficiency were selected for further analysis.

### Differentiating Features Between SC and CI Antibody Repertoires

**Figure 2:**
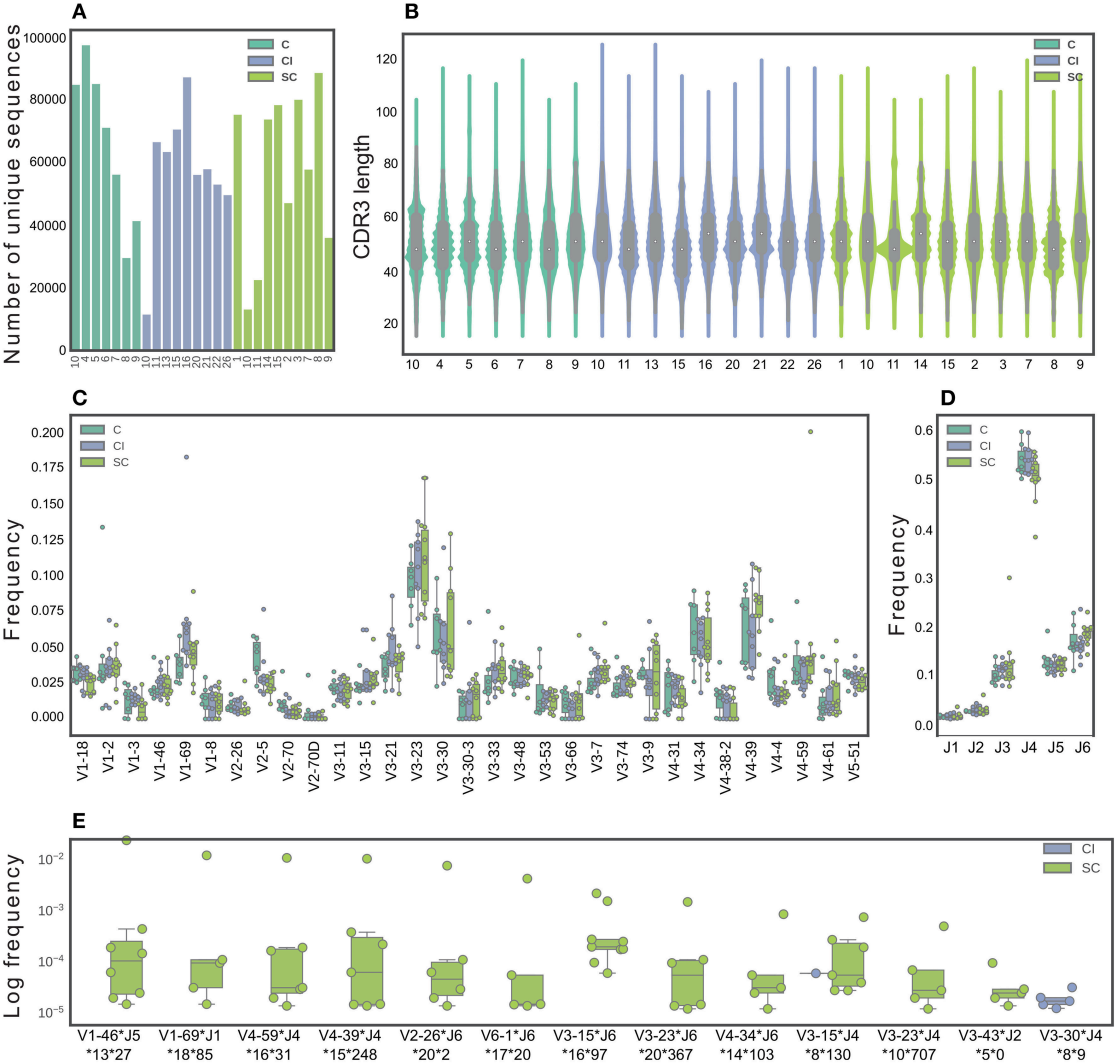
Characterization of B-cell repertoires in SC, CI, and healthy individuals. **(A)** The number of unique sequences per sample after pre-processing. **(B)** The CDR3 length distribution. **(C)** The IGHV gene distribution. Only functional V genes that were in the 15 topmost frequent in at least one sample are shown. **(D)** The IGHJ gene distribution. **(E)** Feature combinations whose abundance differ between the SC and CI groups are presented for sequence clusters grouped by identical IGHV and IGHJ and by high CDR3 similarity, which were significantly more abundant in either SC or CI cohort (|#samples_SC_-#samples_CI_| >3 samples). Sequence logos CDR3 of these clusters are presented in Supplementary Figure 4.

Previous studies suggested defining a successful immune response to HCV by studying SC vs. CI (8, 9, 13, 26). However, a deep insight into these responses is lacking. Here, we sought to use high-resolution technologies that will significantly increase the number of screened samples and the screening depth of each sample. We sequenced antibody repertoires from 28 individuals; among these are 10 HCV CI, 11 SC that displayed the highest neutralization efficiency as described above (Supplementary Figure 2B), and 7 healthy control samples. We identified 10^4^-10^5^ unique full-length heavy chain sequences for each sample (Figure 2A).

To identify features in B-cell repertoires that are unique to CI or SC HCV infections, we evaluated the usage frequency of each V and J gene segment, the CDR3 length, as well as the mutation frequencies across the V genes. Sequences were grouped by their V gene, J gene, and CDR3 length, clustered by genetic distance, and the frequencies within and between the clinical groups were compared. We did not observe significant differences in CDR3 length, V, and J gene distributions between the clinical groups (Figures 2B–D). V-J gene combinations, as well as V-J-CDR3 length also did not yield significant results. We performed a similar analysis for β chains of TCRs from the same individual groups (Supplementary Figure 3A), and did not observe differences in CDR3 length, V, and J gene usage between SC and CI clinical groups (Supplementary Figures 3B–D).

We next sought to explore the possibility that clusters of similar antibody sequences are enriched in either SC or CI groups. To this end, we grouped the antibody sequences by V-J-CDR3 similarity. We identified 337 clusters that are different between the clinical groups by more than four samples. Of these, 165 clusters were enriched in SC samples and 172 clusters were enriched in CI samples. To narrow down the list of candidate clusters for classification, we increased the threshold for calling a cluster enriched, from four samples to five. Using this higher threshold, we identified 13 enriched clusters. Of these, 11 clusters were unique to SC, and one was unique to CI (Figure 2E and Supplementary Figure 4)

To evaluate the mutation frequencies between the clinical groups, we first subdivided the sequences into IgM, IgD, IgG, or IgA isotypes. No significant differences in the frequencies of the different isotypes were observed between the clinical groups (Supplementary Figure 5A). Supplementary Figure 5B displays a violin plot comparing the distribution of somatic mutation frequencies across IgA, IgD, IgG, and IgM. As expected, higher mutation numbers were observed in the IgG and IgA isotypes, compared with the IgM and IgD isotypes. No significant differences were observed in mutation numbers within each isotype between the clinical groups (Supplementary Figure 5C). We also compared mutation numbers for each isotype across V genes between the clinical groups. Interestingly, 14 isotype-specific V genes were significantly different when comparing the clinical groups (Supplementary Figure 5D). Of these, four displayed higher mutation numbers in SC than in CI, including IGHV3-53, IGHV2-70, IGHV1-8, and IGHV3-33. The remaining ten V genes displayed lower mutation numbers in SC than in CI.

### A Machine Learning Model Predicts Clinical Outcomes Based on the Antibody Repertoire

To determine whether a combination of features, rather than one at a time, would provide better insight into the antibody sequences that participate in the response to HCV, we used a machine learning approach, which predicts the clinical group based on a combination of features. This approach can be utilized not only as a prediction model; it can also be used as a tool to identify significant features that did not arise in the single-feature analysis.

For feature selection, we calculated frequency per sample for each cluster of sequences. To avoid false clusters that may occur due to grouping of several erroneous sequences with correct ones, we removed rare clusters that appeared at low frequencies or in fewer than four samples. Then, we left out two samples as a test set, and we trained the model on the remaining samples.

We applied a random forest model to extract the best 18 clusters (equal to the size of the training set), followed by logistic regression on the selected clusters to generate the prediction model. Finally, we applied the model to the remaining two samples and calculated their accuracy. The process of sampling and training was repeated 100 times, to ensure that the model was not biased toward specific samples.

**Figure 3:**
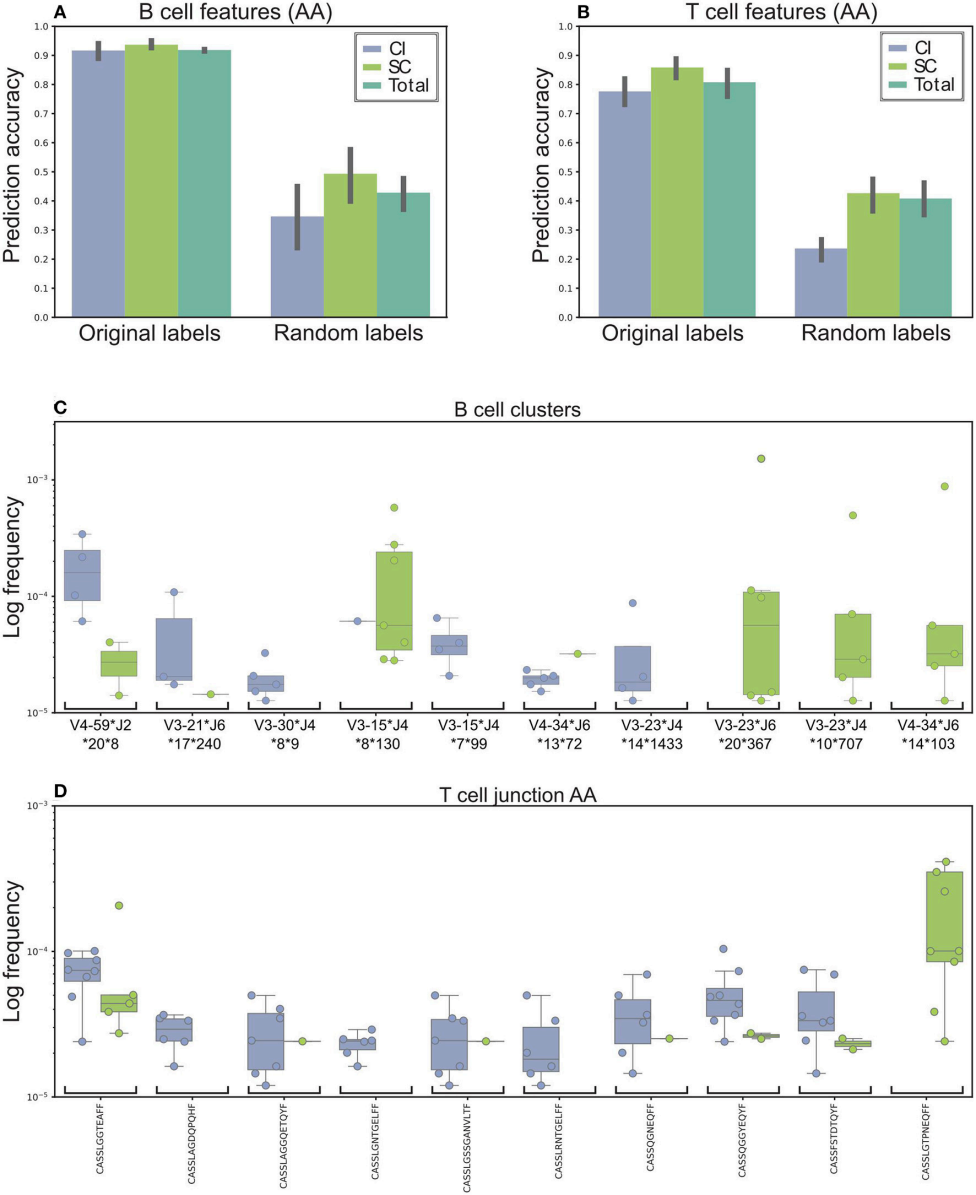
Machine learning model used to stratify between SC and CI. **(A)** Accuracy was based on the B cells' repertoire. Original labels represent clustered sequences by identical IGHV and IGHJ and the high similarity of the CDR3 amino acid sequence. For validation purposes, the model was trained and applied on randomly labeled data. **(B)** Prediction model based on the T cells' repertoire. The training for the T-cell repertoires model is very similar to the B-cell model, except that the data were clustered solely by CDR3 amino acid identity. **(C,D)** The top 10 clusters used by the model to stratify between the cohorts. **(C)** In B-cell clusters. **(D)** In T-cell clusters. Sequence logos of the CDR3 of the B cell clusters are presented in Supplementary Figure 6.

The final predication results, summarized in Figure 3, indicate 91% accuracy of the prediction. As a control, when we randomly shuffled the clinical groups and trained our model, the prediction rates were 49 and 35% for the SC and CI groups, respectively (Figures 3A,B for T cells), suggesting that we did not achieve the high accuracy predictions due to over fitting or another random bias of any specific sample. Therefore, we identified sequence clusters that can accurately stratify between the SC and CI samples (termed “stratifying clusters”). Of the 10 best clusters (Figure 3C; Supplementary Figure 6), four (IGHV3-15^*^IGHJ4^*^8^**^130, IGHV4-34^*^IGHJ6^*^14^**^103, IGHV3-23^*^IGHJ4^*^10^**^707, and IGHV3-23^*^IGHJ6^*^20^**^367) were also previously found in the single-feature comparisons (Figure 2E).

Possible inaccuracies in multiplexed sample sequencing as a result of rare barcode impurities might cause biases. To overcome this difficulty we determined a strict cutoff. We used only clones in which at most 90% of the sequences originated from one sample. If we had not used any cutoff, the prediction precision would improve by only 2%. Lowering the cutoff to 80% decreases the precision by 13.5%. Still, a high performance of the algorithm.

Training the model for T-cell repertoires was very similar to the one for the B-cell repertoires, except that the data were categorized by identical AA CDR3 sequences. The average accuracy was ~79 and 85% for the SC and CI groups, compared with 50% using shuffled labels (Figure 3B). Of the 10 best CDR3 sequences (Figure 3D), two sequences, CASSTAGQGLTEAFF and CASSLGTPNEQFF, were also found in the single feature comparisons.

### Differentiating the Features of HCV-Specific B-cell Repertoires

**Figure 4:**
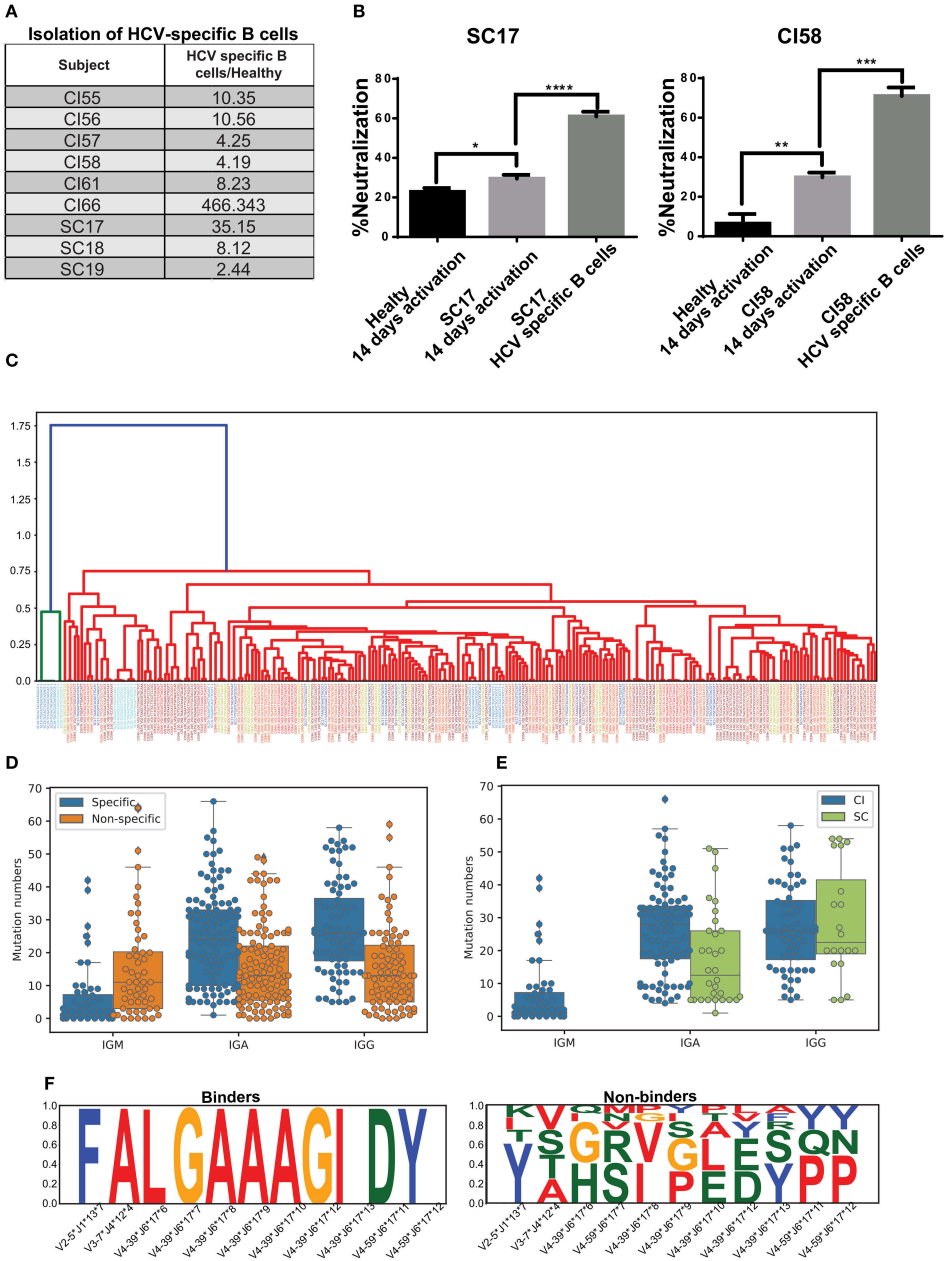
Isolation of HCV-specific B cells from resolved and chronic HCV infection. **(A)** HCV-specific B-cells isolated from six CI and three SC individuals, as compared with control healthy individuals. The fold enrichment of HCV-specific B cells from each sample was calculated compared with the number of B cells isolated from a healthy individual, as demonstrated in Supplementary Figure 7. **(B)** HCVcc-neutralization assays using supernatants of cultured B cells from healthy, SC, and CI samples after two 2 weeks of activation *in vitro*. (*P < 0.03, **P < 0.003, ***P < 0.0001, ****P < 0.00003, *t*-test). **(C)** Dendogram of CDR3s from HCV-specific B cells, generated based on Levenshtein distances. Each color of the CDR3 sequence corresponds to an individual. **(D)** Mutation numbers in IGHV genes in the general repertoire compared with the HCV-specific repertoire. Each specific sequence was randomly matched to a non-specific sequence with the same IGHV and IGHJ genes. The sequences were grouped by isotype and mutations were compared by Mann Whitney test (IGA *p* = 3.488873e-07, IGG *p* = 6.849511e-08, IGM *p* = 3.764229e-04). **(E)** Mutation number in the IGHV genes in the specific repertoire for SC and CI (IGA *p* = 0.000574, IGG *p* = 0.435930). **(F)** Conserved amino acids in CDR3 from the HCV-specific repertoire (binders) compared with the general repertoire (non-binders). For each specific sequence, a non-specific sequence was randomly matched. Sequences were then grouped by IGHV, IGHJ, and CDR3 length. Cases where CDR3 amino acids were very conserved for binder sequences but not for non-binders are shown.

Previous studies reported the frequencies of circulating, antigen-specific B cells in humans of up to 1% of the overall B-cell population (54). Therefore, the polyclonal nature of the immune response may impose significant background noise that interferes with characterizing the HCV-specific immune response. Thus, we sought to isolate HCV-specific B cells and characterize their properties. Here, we have established a novel platform for the *in vitro* propagation and isolation of HCV-specific memory B cells (described in the Materials and methods). The HCV E2^+^-specific populations were separated from six CI and three SC individuals and healthy individuals as controls (Figure 4A). The fold enrichment of HCV-specific B cells from each sample was calculated compared to the number of B cells isolated from healthy individuals, as demonstrated in Supplementary Figure 7. The fold enrichment of cells isolated from HCV-specific B cells ranged from 2 to 466 (Figure 4A). To validate the enrichment of HCV-specific B cells, the growth media of the cells were used for the HCV-neutralization assay, which displayed higher neutralization in the CI and SC samples compared with healthy controls. Neutralization was further enhanced following separation of HCV-specific B cells (Figure 4B).

The variable regions of the antibody's heavy chains of the HCV-specific B cells were sequenced. First, we evaluated the genomic distance of the VDJ region sequences between the different samples by the Levenshtein distance. Interestingly, some of the most closely related sequences originated from different samples (Figure 4C). This observation implies that similar antibodies convergently evolve in different patients to bind HCV. To compare the repertoire of HCV-specific binding sequences with the total repertoire of a given donor, defined here as the “general repertoire,” we searched for sequences in the general repertoire that are similar to the specific binders. Similarity was defined as having the same V gene, J gene, and CDR3 sequence that are at least 75% identical at the AA level. In total, we detected 5,447 clusters in the general repertoire that were similar to the HCV-specific repertoire. In the specific repertoire we identified 17 clusters that were enriched in SC samples in the general repertoire, and 15 clusters that were enriched in CI samples in the general repertoire. An enriched cluster was defined as being represented in more than three samples in the cohort, and in addition, the fraction of samples in the cohort representing this cluster out of the total number of samples representing it is larger than 2/3. The lists of these clusters are presented in Supplementary Tables 3,4. A comparison between these two lists reveals that except for the V-J combination IGHV3-33^*^IGHJ4, which is abundant in both lists, different HCV-binding clusters are enriched in the two clinical groups.

Another feature that we have analyzed in the general repertoire, compared to the specific repertoire, is mutability. Against each specific sequence, one non-specific sequence was randomly sampled from the general repertoire. The sampled sequence contained the same V and J gene as the corresponding specific sequence. Then, sequences were grouped by isotype, and mutation numbers in the V gene were compared. Both for IgA and IgG, we detected significantly higher mutation numbers in specific compared with non-specific repertoires. For IgM, however, we observed an opposite trend (Mann Whitney test, IGA *p* = 3.488873e-07, IGG *p* = 6.849511e-08, IGM *p* = 3.764229e-04) (Figure 4D). This might result from the long infection period of the chronic HCV patients.

We then evaluated the mutation number in the HCV-specific repertoire in SC compared with CI. All specific sequences of SC samples were unified into one bulk, and CI samples were unified in a second bulk. Then, the sequences were grouped by isotype and the mutation numbers in the V genes were compared. The number of mutations in the SC-specific repertoire bulk was lower than that in the CI-specific repertoire (Figure 4E). This is expected, as in CI the B cells have been through longer and repeated rounds of somatic hypermutation process which is consistent with a chronic situation that allowed the accumulation of mutations, compared with the short period of infection in SC.

The heavy chain CDR3 is the most diverse region in the antibody sequences. Therefore, conservation of AAs in this region can highlight positions that are important for antigen binding. Here we searched for conserved AAs in the CDR3 region in the HCV-specific repertoire compared to the general repertoire. Against each binder sequence, we selected a random sequence with identical V, J, and CDR3 lengths from the general repertoires, defined as non-binder. Then, amino acids that were conserved in binder sequences but not in non-binders were selected. We identified four combinations of V, J, and CDR3 lengths containing differentially conserved AAs in CDR3 (Figure 4F). Interestingly, IGHV4-39–IGHJ6–17 contained a stretch of seven conserved residues in CDR3 and was observed in three different samples (CI56H, CI57H, and CI59H). These results imply that clones evolved independently in different subjects and converged to similar CDR3 AA patterns.

### Identifying Binder Antibody Sequences Associated With HCV Infection Clearance

We next sought to construct antibodies that are associated with infection clearance, and to explore their properties. One limitation of constructing mAbs directly from bulk repertoire analysis is the pairing of heavy and light chains. We applied an approach for matching heavy with light chains, by constructing a phage display antibody library. These antibodies contain the variable regions of both heavy and light chains as a single chain (scFv), and thus enable the design of full antibodies (55).

**Figure 5:**
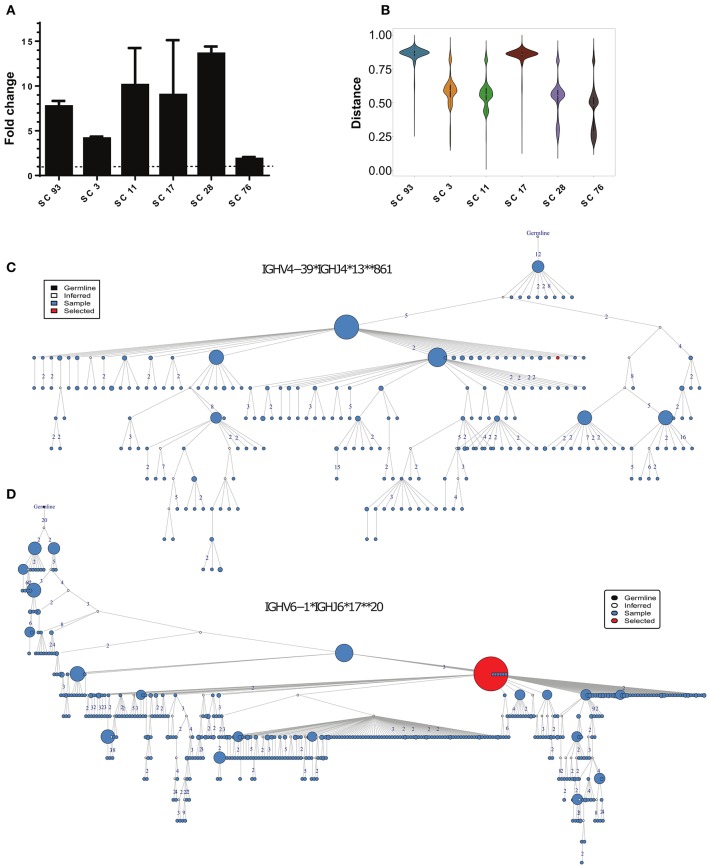
Identification of HCV-specific antibody sequences associated with HCV infection clearance. **(A)** Binding of the phage-displayed antibodies to the rE2 protein (5 μg/ml) by ELISA. Each bar indicates the mean fold change ± SD in the OD compared with BSA binding, from three independent experiments. **(B)** Violin plot of the distances between HCV-specific sequences and the healthy, CI and SC repertoires. **(C,D)** Phylogenetic trees of the two closest clusters to scFv SC11 **(C)**, and SC28 **(D)**.

Since we specifically focused on nAbs associated with HCV clearance, we have constructed a phage display antibody library from a source of pooled PBMCs obtained from 10 SC individuals (Supplementary Table 1). The scFv library was constructed by amplification of the VH and VL genes separately, and then their combinatorial assembly and cloning into a phagemid vector. In total, we obtained a library of 6^*^10^7^ individual scFvs. We screened for HCV E2 binders, and identified and validated six different phages that displayed 2- to 15-fold binding to rE2 compared with BSA as background (Figure 5A). We then identified clusters of sequences from the general repertoire that were similar to the isolated scFv sequences, and selected the closest sequence to each scFv (Figure 5B).

We searched for candidates for constructing full-length antibodies from these six scFvs. We decided to focus on scFv SC11 and SC28, since they showed the highest binding to HCV E2 protein (Figure 5A) and were the most similar to the SC general repertoires (Figure 5B;Supplementary Figure 8). The closest cluster to scFv SC28 was IGHV4-39^*^IGHJ4^*^13^*^861, which was detected in the repertoires of four out of nine SC samples, and the closest cluster to scFv SC11 was IGHV6-1^*^IGHJ6^*^17^**^20, which was detected in repertoires of five out of nine SC samples (Figure 2E). Both clusters were not detected in CI repertoires. Cluster IGHV6-1^*^IGHJ6^*^17^**^20 was also enriched in the HCV-specific repertoire (Supplementary Table 4). Lineage trees revealed that the closest sequences to SC11 and SC28 are positioned relatively high in the tree (Figures 5C,D), suggesting that these sequences appeared earlier during the infection. We therefore selected scFvs SC11 and SC28 as candidates for constructing full-length antibodies and characterizing their properties.

### Construction of Broadly Neutralizing Antibodies Associated With HCV Infection Clearance

**Figure 6:**
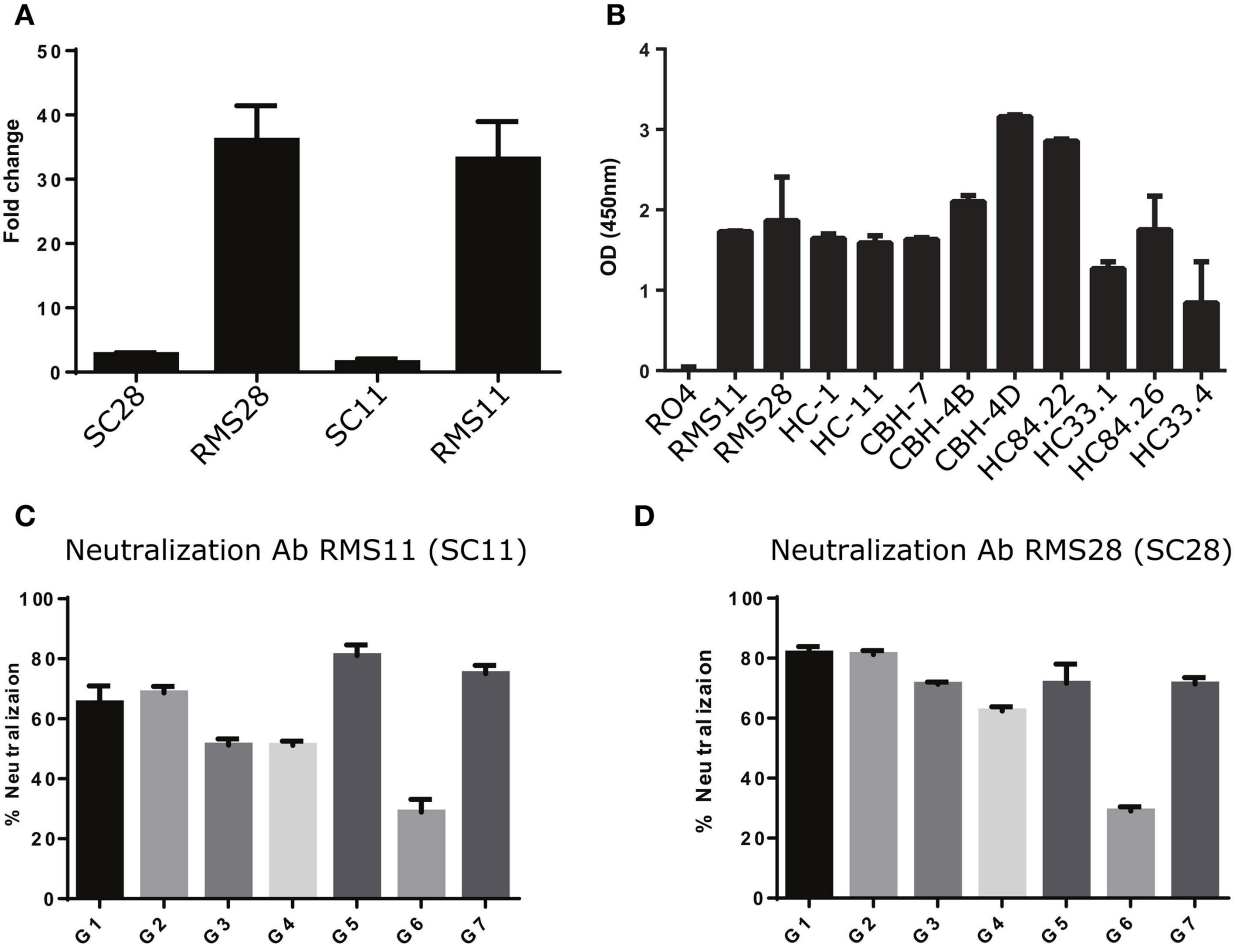
Construction and characterization of antibodies correlated with infection clearance. **(A)** Binding of antibodies RMS28 and RMS11 to the rE2 protein (5 μg/ml) compared with the phage display antibodies SC28 and SC11 by ELISA, using 16 μg/ml Ab. Each bar indicates the mean fold change ±SD in binding, compared with BSA, from three independent experiments. **(B)** Binding of antibodies RMS11 and RMS28 to the rE2 protein (5 μg/ml), compared with a well-defined panel of nAbs and a non-specific control antibody RO4 by ELISA, using 16 μg/ml Ab. Presented are mean OD (450 nm) values ±SD, from three independent experiments. **(C,D)** HCVcc neutralization assays were carried out with genotypes G1-G7 using 20 μg/ml of antibodies RMS11 **(C)** and RMS28 **(D)**. The percent neutralization was calculated as the percent reduction in FFU compared with virus incubated with an irrelevant control antibody (RO4). Presented are means of % neutralization ±SD from three independent experiments.

We constructed and produced full-length antibodies from scFvs SC11 and SC28. In addition, we constructed and produced full-length antibodies with identical light chains, but the heavy chains were replaced with one of the nearest sequences to the heavy chains of scFv SC11 and scFv SC28 from the general repertoires (RMS11 and RMS28, respectively). We evaluated the binding specificities of these four antibodies to HCV rE2 protein. We observed more than 35-fold higher binding signals in antibodies RMS11 and RMS28 than with antibodies SC11 and SC28 (Figure 6A). To further characterize the binding capacity of RMS11 and RMS28, we compared the binding of these antibodies to a well-characterized panel of mAbs, including CBH-4B, CBH-4D, HC-1, HC-11, CBH-7, HC84.22, HC84.26, HC33.1, and HC33.4, which are representative E2 antigenic domain A-E antibodies [(12) and reviewed in (20, 21)]. ELISA results with rE2 protein indicated binding capacity of RMS11 and RMS28 comparable to the well-defined panel (Figure 6B). To evaluate neutralization breadth, we performed neutralization assays with these antibodies across all HCV genotypes using a panel of infectious HCVcc containing envelope proteins from HCV genotypes 1–7 (41). The percent neutralization was calculated as the percent reduction in FFU compared with virus incubated with an irrelevant control antibody RO4 (56–59). Antibodies RMS11 and RMS28 efficiently neutralized all seven HCV genotypes, including genotype three which was less efficiently neutralized by previous panels of HCV antibodies including a recent SC panel (26), pointing out their exceptionally high neutralization breadth (Figures 6C,D).

## Discussion

This study provides the first in-depth analysis of HCV-specific immune response and identifies features that correlate with infection outcome. The landscapes of B- and T-cell repertoires, including usage of specific V and J genes, CDR3 lengths, and mutation numbers, did not significantly differ between the SC and CI groups. The most prominent differences between SC and CI are specific sequence clusters enriched in one of the groups, identified both in the general and in the HCV-specific B-cell repertoires. Strikingly, we found that enrichment of specific clusters in SC or CI is indicative of infection outcome, and with an accuracy of over 90% for B-cell repertoires and 80% for T-cell repertoires. This may have important clinical relevance as well as prognostic value for the outcome of an active infection. In the DAAs era, when the availability of effective HCV therapy is limited by the high costs (2), using the platform we have established may indicate the best clinical decisions for treatment.

Fewer mutations were observed in the general B-cell repertoires compared with HCV-specific B-cell repertoires. In addition, fewer mutations were observed in HCV-specific B-cell repertoires from SC vs. CI. These findings validate a recent study demonstrating that a panel of HCV- nAbs isolated from SC contained a lower number of mutations compared with HCV- nAbs isolated from CI (26). Our findings expand the above results to many HCV-specific sequences from multiple individuals. Moreover, we validated the broad neutralization potential of two of the identified HCV-specific sequences observed in SC. Broadly nAbs were suggested to be induced in the early stages of infection in SC, whereas CIs were associated with the induction of such antibodies at later stages. Furthermore, CI antibodies require higher mutation numbers to achieve broad neutralization to the variable quasi-species population of viruses that evolved in these later stages. Therefore, it has been suggested that bnAbs with a relatively low number of mutations are associated with viral clearance (26). In contrast to HCV, in the case of HIV infection, bnAbs require high mutation numbers and many years to evolve (60–62). Indeed, the ability to provoke broad neutralization with low mutation numbers in HCV infection is translated to approximately 30% SCs, compared with none in HIV infections (7). Here, we show that HCV-specific antibodies in SC are characterized by not only low mutation numbers and high neutralization breath compared with antibodies in CI, but also that the context of these differences is within different clusters of sequences between the two clinical groups. These findings point to the conclusion that the immune response to HCV infection provoked in SC is largely different from that provoked in CI. Therefore, we provide the first evidence that the nature of the immune response is associated with infection outcome and not only with the timing of the appearance of bnAbs, as was suggested previously (13, 26).

It will be most interesting to determine whether antibodies that are unique to SC are also characterized by binding to distinct epitopes. Similar epitope specificities were demonstrated for the recent panel of nAbs isolated from SC infections (26). Still, it has been suggested that nAbs with distinct epitope specificities do exist but remain to be discovered (12). Discovering novel epitopes will point to new mechanisms driving infection outcome.

The construction of two antibodies, identified by combining phage display antibody library technology and antibody repertoires of SC, yielded HCV- nAbs with exceptional potential of broad neutralization breadth. Our finding that specific clusters are specific for clearance of HCV infection, whereas others are specific for progression to chronic infection, demonstrates that similar antibodies convergently evolved in different individuals. Identifying fractions of these clusters in the HCV-specific repertoire validates that they are provoked in response to the infection and consequently likely bind the virus. Sharing identical CDR3 sequences by different individuals was suggested to be very rare (63), although such immunological signatures were reported in viral-specific responses (33). These discoveries raise the intriguing question of what governs these pronounced similarities in the antibody's response to HCV in different individuals, which is indicative of infection outcome.

Previous publications have suggested that VH1-69 is enriched in clusters identified in both SC and CI, based on isolating a panel of HCV-nAbs (25, 26, 64). However, our high-resolution approach, which provides a wide overview of the general repertoire and HCV-specific repertoire, demonstrates that this gene is more abundant in CI than in SC repertoires, and that it is not enriched in HCV-specific repertoires.

In summary, this study provides a novel high-resolution insight into the nature of the HCV-specific immune response, and demonstrates for the first time that the outcome of infection is determined by the unique features of the immune response. Our innovative approach combines antibody repertoire analysis and antibody engineering tools that provide the high sensitivity necessary to identify antibody sequences enriched in SC vs. CI infections, and use this information to produce full antibodies. Identifying the epitopes of these antibodies may provide translational information for designing a rational prophylactic vaccine. In addition, passive immunization with combinations of mAbs possessing well-defined epitope specificities may overcome virus resistance (65), confer a prophylactic effect, such as in liver transplantation (66), where re-infection of the transplant is rapid (67), and may also prove effective in treating existing HCV infections (24). From a more general point of view, the in-depth analysis of immune repertoires demonstrated here may open a world of possibilities for advancing monoclonal antibody discovery and engineering strategies, which bear many potential clinical implications.

## Author Contributions

MG-T and GY: Conceptualization. MG-T, GY, SiE, OS, ShE, AD, CC, FV, PP, and RH: Methodology. GY and OS: Software. MG-T, GY, SiE, OS, ShE, AD, RT, and YW: Formal Analysis. MG-T, GY, SiE, OS, ShE, AD, PP, RH, and YW: Investigation. MG-T, GY, AN, and MB: Resources. MG-T, GY, OS, and PP: Writing—Original Draft. MG-T, GY, PP, OS, SiE, and RT: Writing—Review & Editing. MG-T and GY: Supervision. MG-T and GY: Funding Acquisition.

### Conflict of Interest Statement

The authors declare that the research was conducted in the absence of any commercial or financial relationships that could be construed as a potential conflict of interest.

## Supplementary Material

Supplementary Figure 1 Enrichment of the HCV-specific B-cell population *in vitro*. For the *in vitro* proliferation of B cells, CD19^+^ cells were isolated from PBMCs of healthy donors using a FACS sorter. Isolated B cells were labeled with CFSE, cultured in the presence of IL2, IL21, and feeder irradiated 3T3-msCD40L cells, and activated with a pool of positive peptides for 8 days. **(A)** CFSE profile of CD19^+^ B cells. CFSE fading (right panel) indicates the proliferation of the activated culture, compared with the non-activated culture (left panel). **(B)** Evaluating the proliferation of memory B cells. In the activated culture, 23% of the population consists of memory B cells that are positive for CD27^+^ (right panel), compared with very low numbers of CD27^+^ cells in the non-activated culture (left panel). **(C)** Evaluating the ability of B cells to differentiate and produce IgGs. The concentrations of IgG secreted to the culture medium 3 or 8 days following B-cell activation were measured by ELISA. (^**^*P* < 0.003, ^***^*P* < 0.0003, ^****^*P* < 0.00003). Presented are means ±SD from three independent experiments.

Supplementary Figure 2 Characterization of sera from HCV-infected individuals. **(A)** HCV antibodies binding to rE2 protein (0.5 μg/ml) performed with 1:1,000 diluted sera of CI (*n* = 52) and SC (*n* = 18) by ELISA. Each dot represents a patient. The background of the binding to BSA was subtracted from all samples. Presented are mean OD (450 nm) values from three independent experiments. **(B)** The HCVcc neutralization assays were performed with 1:1,000 diluted sera of CI (*n* = 52) and SC (*n* = 18) to screen for antibodies that can neutralize HCV infection. The Y axis shows the percentage of neutralization capacity compared with neutralization by sera from a healthy control. Each dot represents the mean neutralization for a patient, from three independent experiments. **(C,D)** Characterizing HCV binding and neutralizing in sera obtained from two patients (CI21 and CI22) before and after anti-HCV treatment and following SVR by ELISA (with 0.5 μg/ml rE2 protein and 1:1,000 diluted sera) **(C)** and by the HCVcc neutralization assay (with 1:1,000 diluted sera) **(D)**. The HCV-cured blood samples were collected from 6 months to 1 year after achieving a sustained virological response. ^**^*P* < 0.003, ^***^*P* < 0.0003. Presented are means ±SD from three independent experiments.

Supplementary Figure 3 General characterization of T-cell repertoires of resolved and chronic HCV infection. **(A)** The number of sequences per sample after pre-processing. **(B)** TRBJ gene usage, colored by clinical group. **(C)** CDR3 length distribution per sample, colored by clinical group. **(D)** TRBV gene usage, colored by clinical group.

Supplementary Figure 4 CDR3 from the SC and CI abundant B cells clusters. Sequence logos of the overall AA composition of the CDR3s in copious clusters. The individual abundance of these clusters is shown in Figure 2E.

Supplementary Figure 5 IGHV mutation characterization in SC and CI infections. **(A)** Isotype usage distribution. **(B)** IGHV mutation distribution, per isotype. **(C)** IGHV mutation distribution per isotype per cohort. **(D)** IGHV mutation distribution per isotype per cohort per IGHV gene. Only statistically significant combinations are shown (*P* < 0.05, *t*-test).

Supplementary Figure 6 CDR3 from the SC and CI B cells clusters used for the Logistic Regression model. Sequence logos of the overall AA composition across the CDR3s in the top 10 clusters used by the model to stratify between the cohorts. The individual abundance of these clusters is shown in Figure 3C.

Supplementary Figure 7 Isolation of HCV-specific B cells from SC, CI, and healthy donors by FACS. CD19^+^ B cells from SC17 and CI58 were grown with feeder-irradiated 3T3-msCD40L cells and activated with 5 μg/ml rE2 protein, IL2, and IL21 for 13–14 days. After 14 days, activated B cells were incubated with 5 μg/ml rE2 and stained with CD19-PE, CD27-BV421, and tagged rE2 (anti-cMyc, alexa fluor 633). Viable, CD19^+^, CD27^+^, and HCsAg^+^ were isolated by FACS. The gating region is shown as a black rectangular.

Supplementary Figure 8 The distance between scFv antibody sequences and clusters from B-cell repertoires of SC and CI infection. Each dot represents the average distances between the scFv antibody sequence and the 10 closest sequences (by VDJ, amino acid sequence) of the B-cell repertoire from healthy controls (light blue), CI (blue), and SC (green). The lower the distance, the more similar is the scFv antibody sequence.

Supplementary Table 1 Features of studied subjects.

Supplementary Table 2 List of primers.

Supplementary Table 3 Clones detected in HCV-specific B cell repertoire and enriched in CI.

Supplementary Table 4 Clones detected in HCV-specific B cell repertoire and enriched in SC samples.
