# Supplementary figures and images for: Antibody Repertoire Analysis of Hepatitis C Virus Infections Identifies Immune Signatures Associated With Spontaneous Clearance
Source: Front Immunol. 2018 Dec 21;9:3004. doi: 10.3389/fimmu.2018.03004 (PMC6308210; doi:10.3389/fimmu.2018.03004)

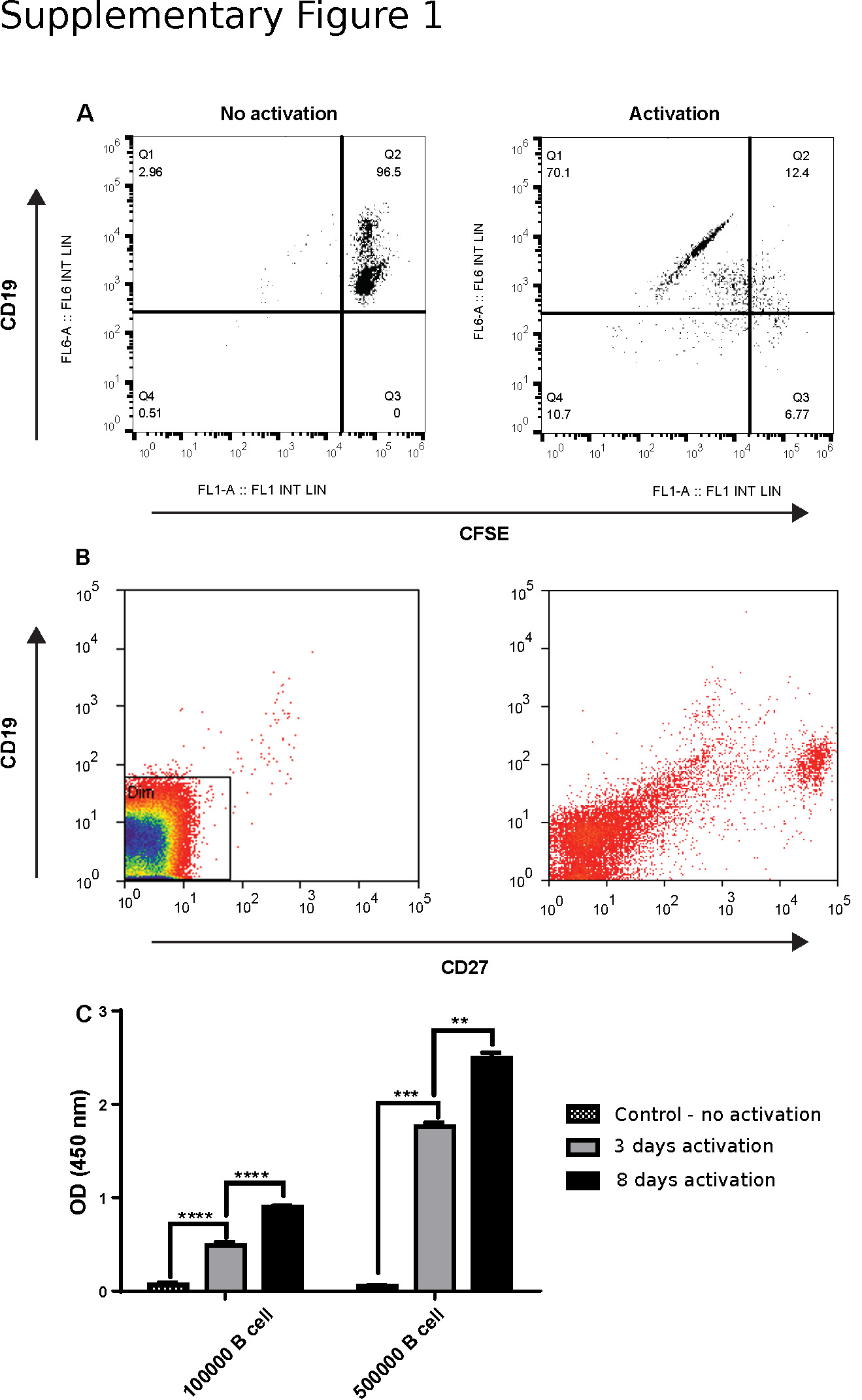

Supplement: Supplementary Figure 1 — Enrichment of the HCV-specific B-cell population in vitro. For the in vitro proliferation of B cells, CD19+ cells were isolated from PBMCs of healthy donors using a FACS sorter. Isolated B cells were labeled with CFSE, cultured in the presence of IL2, IL21, and feeder irradiated 3T3-msCD40L cells, and activated with a pool of positive peptides for 8 days. (A) CFSE profile of CD19+ B cells. CFSE fading (right panel) indicates the proliferation of the activated culture, compared with the non-activated culture (left panel). (B) Evaluating the proliferation of memory B cells. In the activated culture, 23% of the population consists of memory B cells that are positive for CD27+ (right panel), compared with very low numbers of CD27+ cells in the non-activated culture (left panel). (C) Evaluating the ability of B cells to differentiate and produce IgGs. The concentrations of IgG secreted to the culture medium 3 or 8 days following B-cell activation were measured by ELISA. (**P < 0.003, ***P < 0.0003, ****P < 0.00003). Presented are means ±SD from three independent experiments. [file Image_1.JPEG]

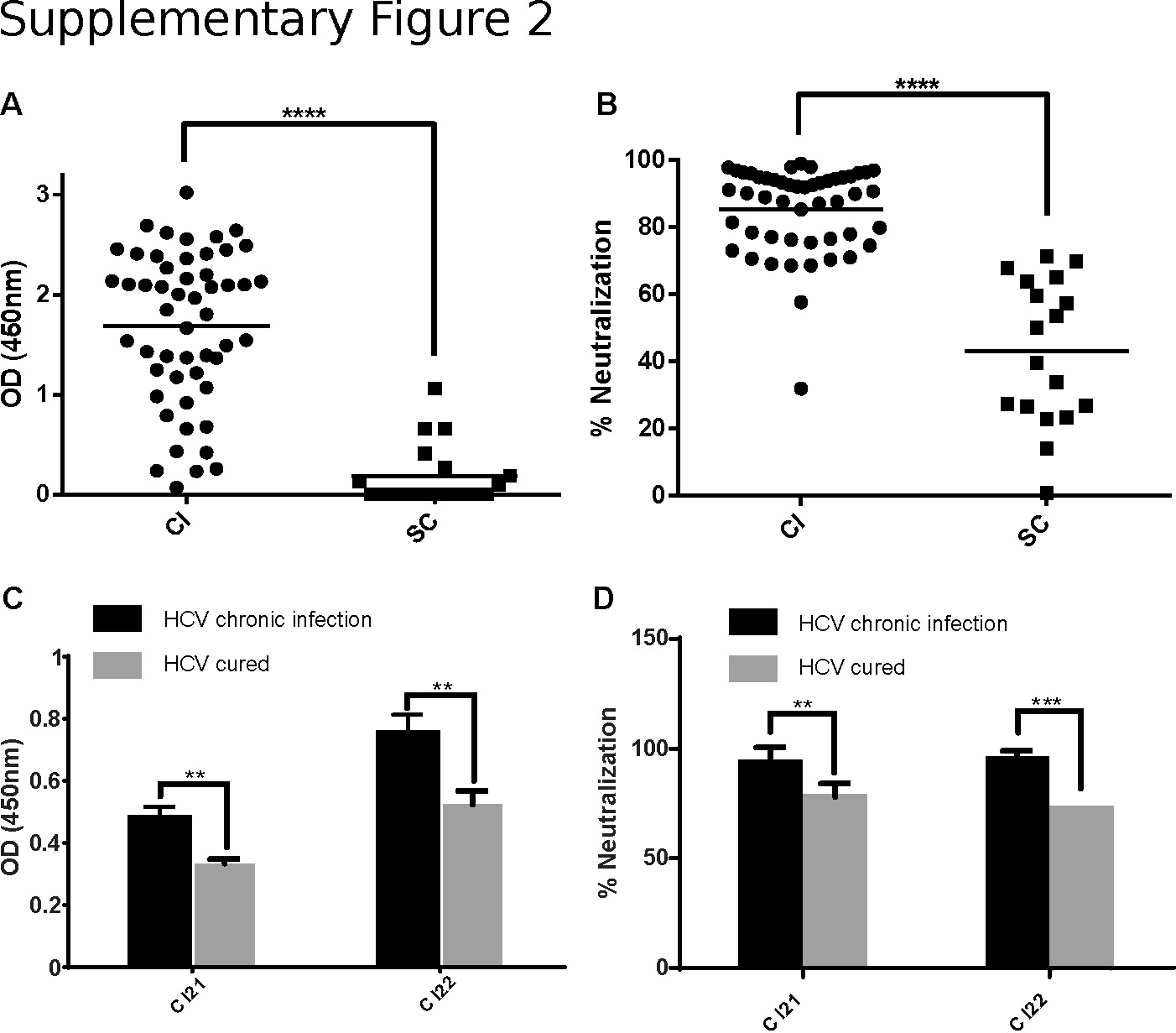

Supplement: Supplementary Figure 2 — Characterization of sera from HCV-infected individuals. (A) HCV antibodies binding to rE2 protein (0.5 μg/ml) performed with 1:1,000 diluted sera of CI (n = 52) and SC (n = 18) by ELISA. Each dot represents a patient. The background of the binding to BSA was subtracted from all samples. Presented are mean OD (450 nm) values from three independent experiments. (B) The HCVcc neutralization assays were performed with 1:1,000 diluted sera of CI (n = 52) and SC (n = 18) to screen for antibodies that can neutralize HCV infection. The Y axis shows the percentage of neutralization capacity compared with neutralization by sera from a healthy control. Each dot represents the mean neutralization for a patient, from three independent experiments. (C,D) Characterizing HCV binding and neutralizing in sera obtained from two patients (CI21 and CI22) before and after anti-HCV treatment and following SVR by ELISA (with 0.5 μg/ml rE2 protein and 1:1,000 diluted sera) (C) and by the HCVcc neutralization assay (with 1:1,000 diluted sera) (D). The HCV-cured blood samples were collected from 6 months to 1 year after achieving a sustained virological response. **P < 0.003, ***P < 0.0003. Presented are means ±SD from three independent experiments. [file Image_2.JPEG]

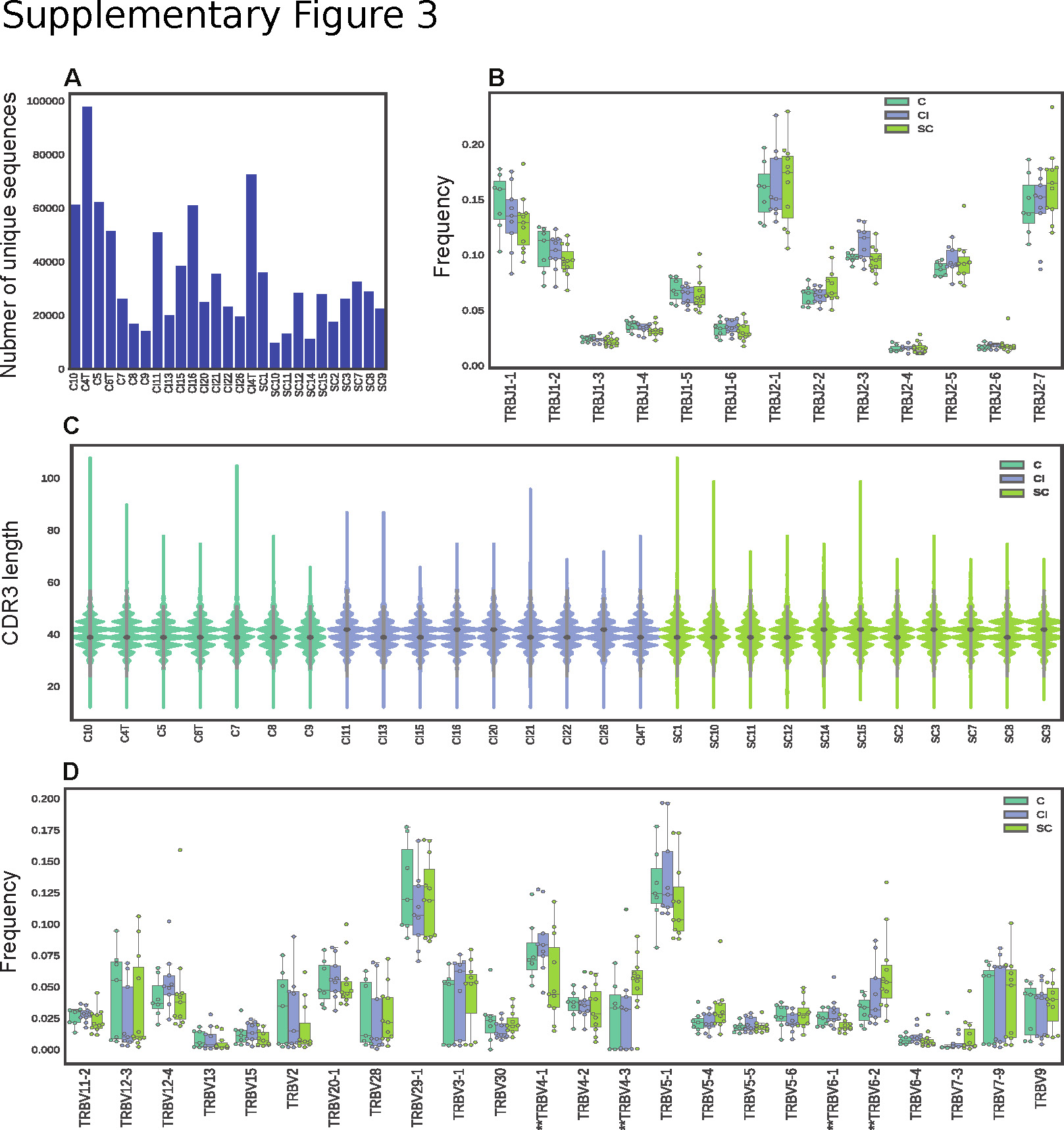

Supplement: Supplementary Figure 3 — General characterization of T-cell repertoires of resolved and chronic HCV infection. (A) The number of sequences per sample after pre-processing. (B) TRBJ gene usage, colored by clinical group. (C) CDR3 length distribution per sample, colored by clinical group. (D) TRBV gene usage, colored by clinical group. [file Image_3.JPEG]

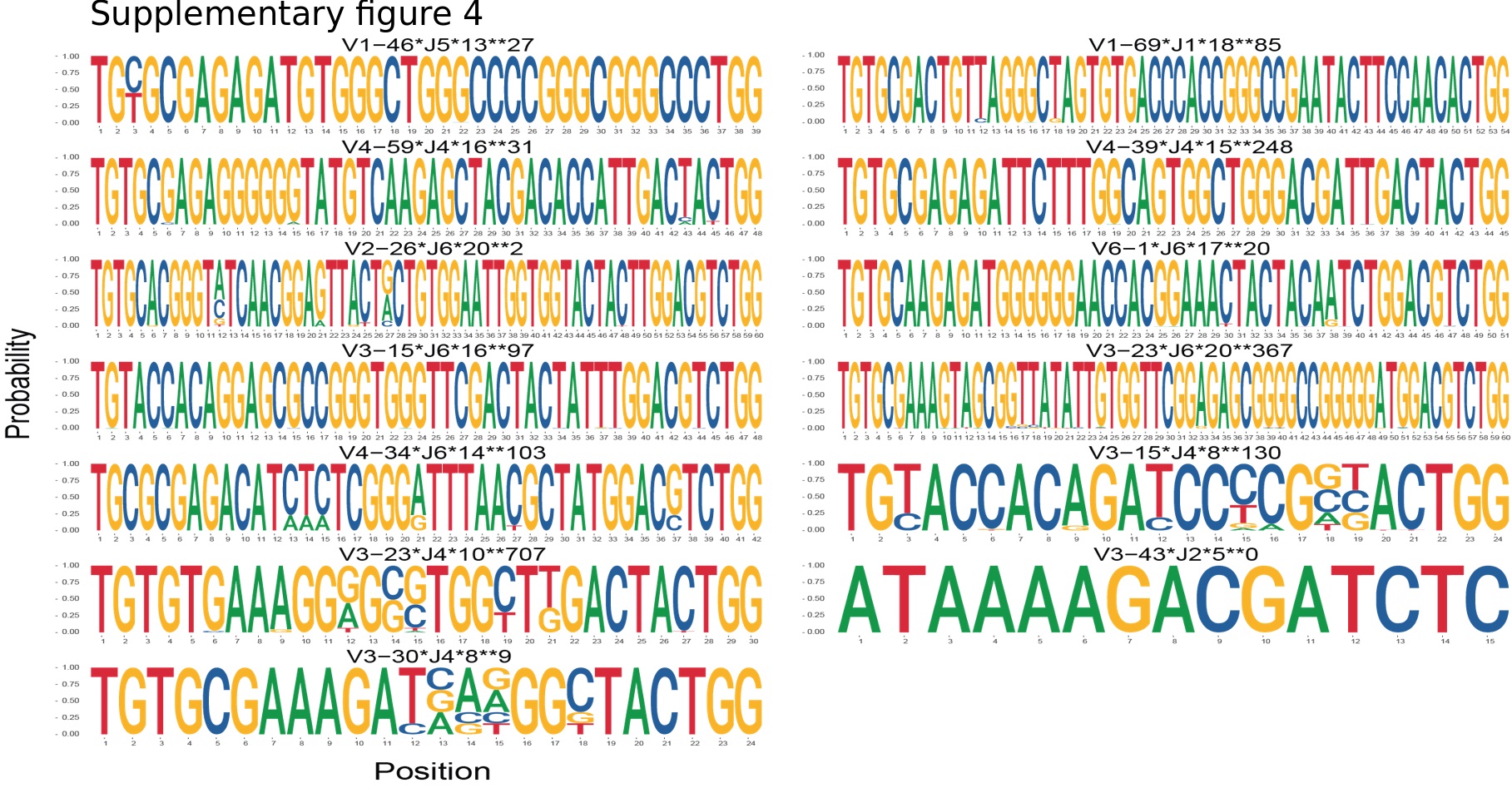

Supplement: Supplementary Figure 4 — CDR3 from the SC and CI abundant B cells clusters. Sequence logos of the overall AA composition of the CDR3s in copious clusters. The individual abundance of these clusters is shown in Figure 2E. [file Image_4.JPEG]

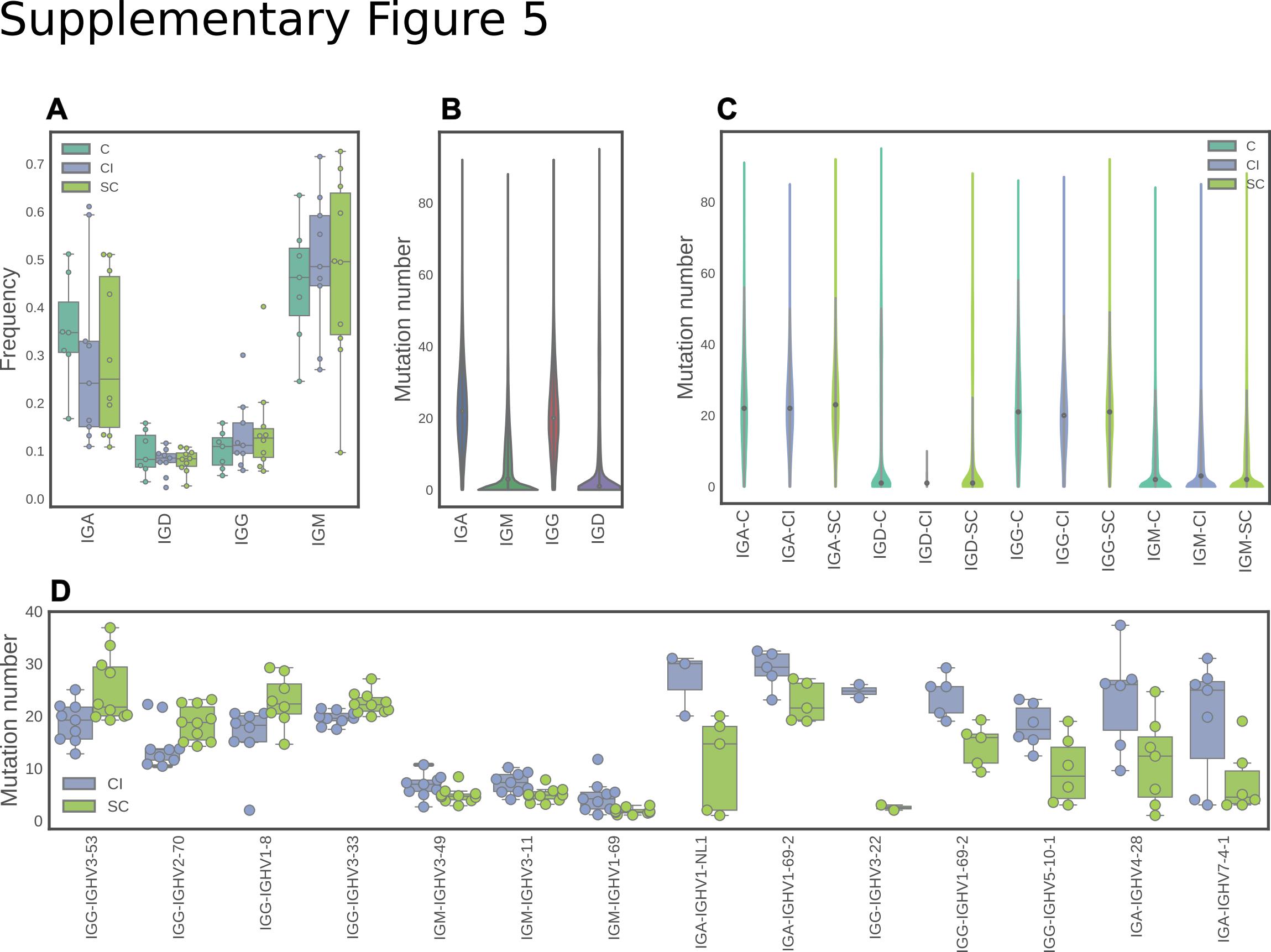

Supplement: Supplementary Figure 5 — IGHV mutation characterization in SC and CI infections. (A) Isotype usage distribution. (B) IGHV mutation distribution, per isotype. (C) IGHV mutation distribution per isotype per cohort. (D) IGHV mutation distribution per isotype per cohort per IGHV gene. Only statistically significant combinations are shown (P < 0.05, t-test). [file Image_5.JPEG]

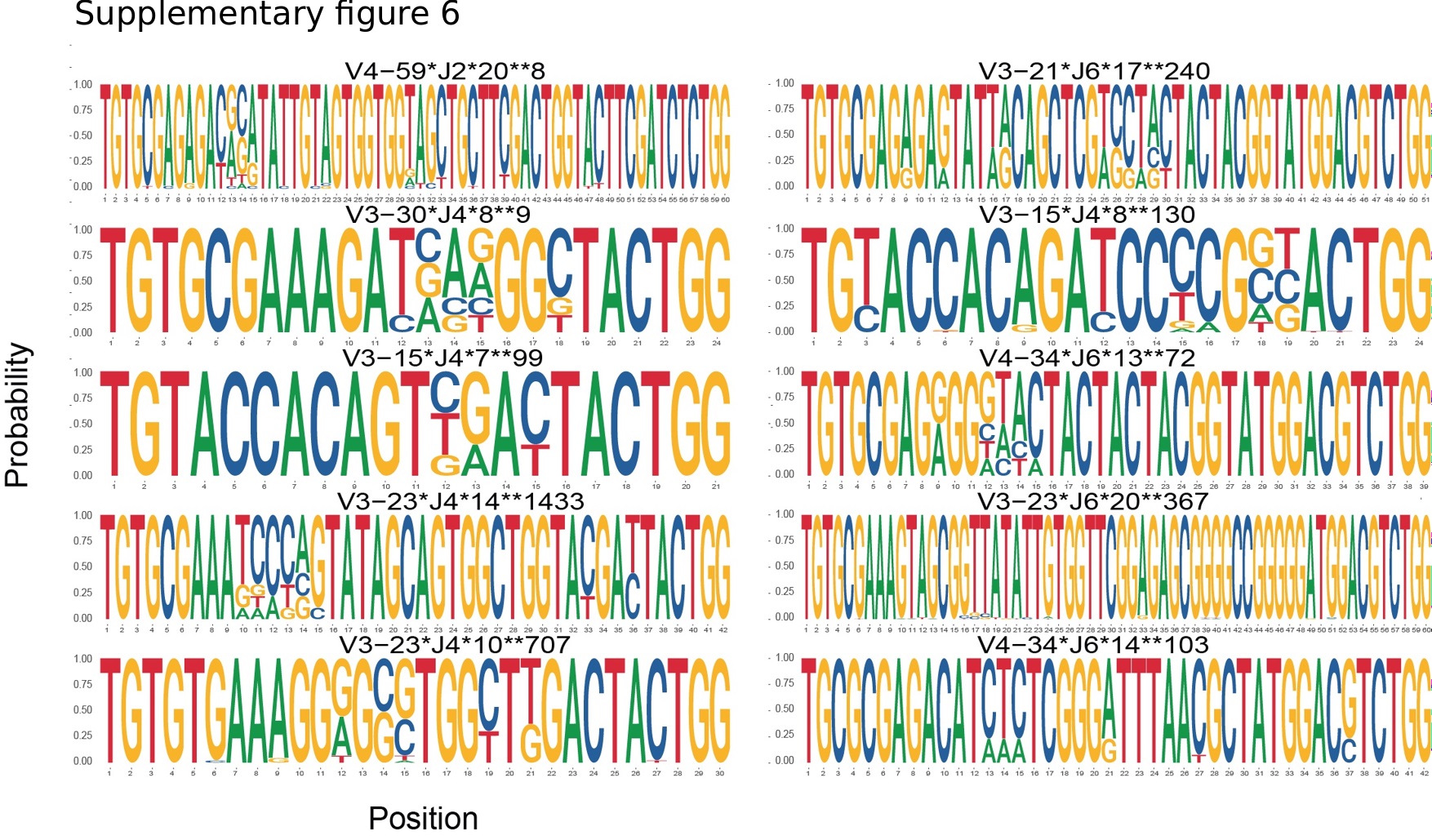

Supplement: Supplementary Figure 6 — CDR3 from the SC and CI B cells clusters used for the Logistic Regression model. Sequence logos of the overall AA composition across the CDR3s in the top 10 clusters used by the model to stratify between the cohorts. The individual abundance of these clusters is shown in Figure 3C. [file Image_6.JPEG]

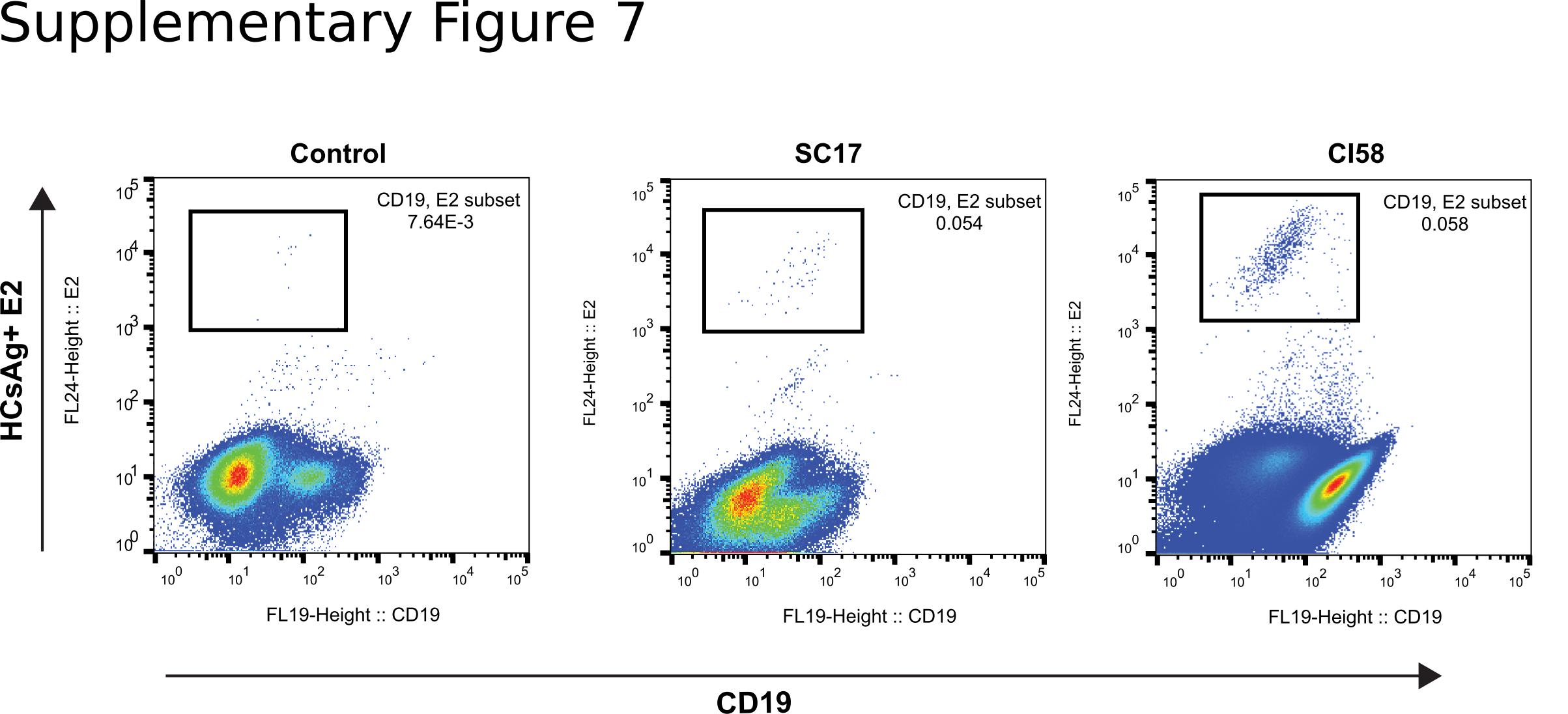

Supplement: Supplementary Figure 7 — Isolation of HCV-specific B cells from SC, CI, and healthy donors by FACS. CD19+ B cells from SC17 and CI58 were grown with feeder-irradiated 3T3-msCD40L cells and activated with 5 μg/ml rE2 protein, IL2, and IL21 for 13–14 days. After 14 days, activated B cells were incubated with 5 μg/ml rE2 and stained with CD19-PE, CD27-BV421, and tagged rE2 (anti-cMyc, alexa fluor 633). Viable, CD19+, CD27+, and HCsAg+ were isolated by FACS. The gating region is shown as a black rectangular. [file Image_7.JPEG]

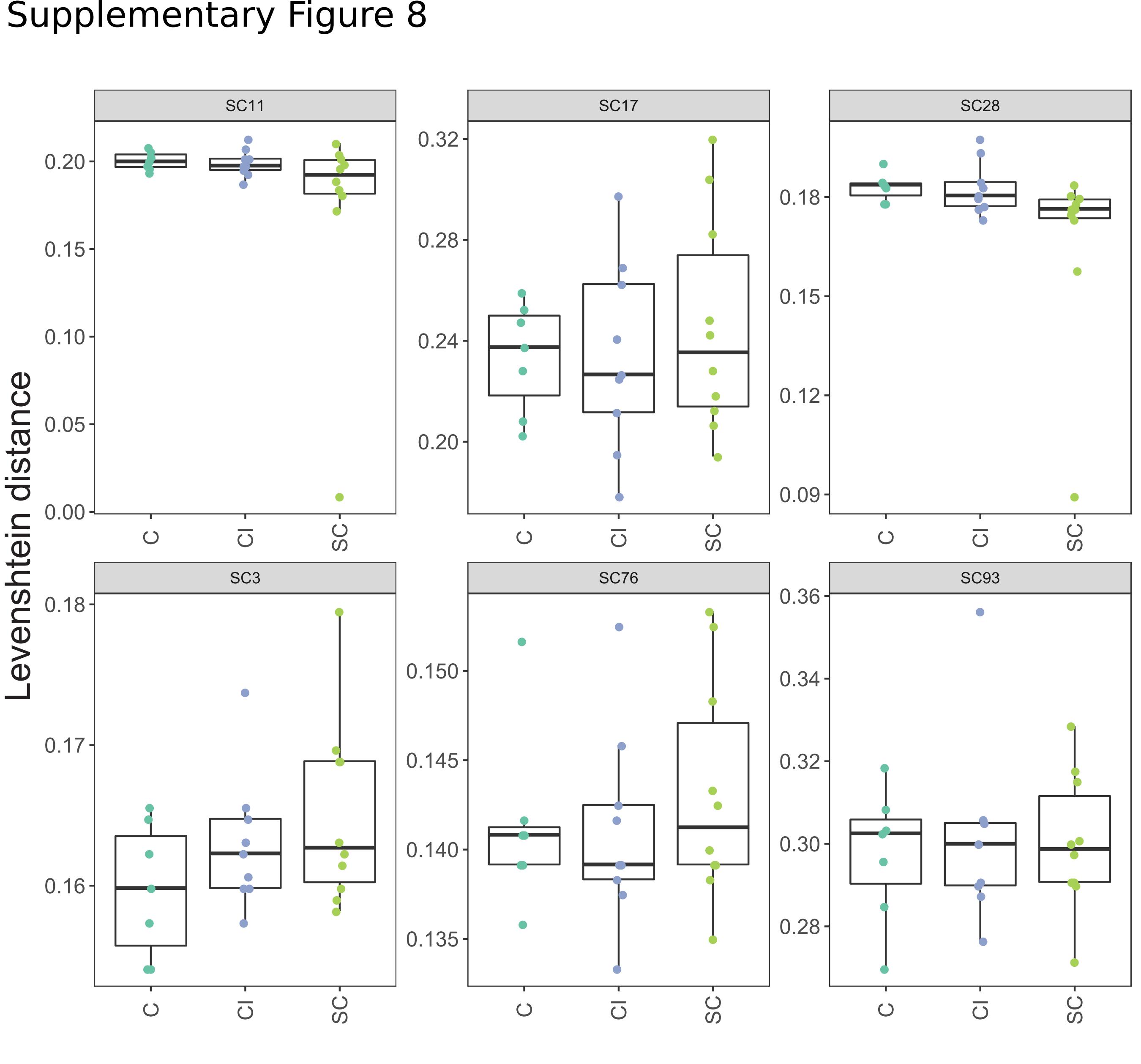

Supplement: Supplementary Figure 8 — The distance between scFv antibody sequences and clusters from B-cell repertoires of SC and CI infection. Each dot represents the average distances between the scFv antibody sequence and the 10 closest sequences (by VDJ, amino acid sequence) of the B-cell repertoire from healthy controls (light blue), CI (blue), and SC (green). The lower the distance, the more similar is the scFv antibody sequence. [file Image_8.JPEG]
