# Supplementary material for: Antibody Repertoire Analysis of Hepatitis C Virus Infections Identifies Immune Signatures Associated With Spontaneous Clearance
Source: Front Immunol. 2018 Dec 21;9:3004. doi: 10.3389/fimmu.2018.03004 (PMC6308210; doi:10.3389/fimmu.2018.03004)
Supplement: Supplementary Table 1 — Features of studied subjects. [file Table_1.DOCX]

**Supplementary table 1. Features of studied subjects**

| **Analysis**  **(General repertoire/**  **specific repertoire/**  **Library)** | **HCV genotype** | **Sex** | **Sample ID** | **Chronic HCV (CI)/**  **Cleared HCV (SC)/**  **Control (C)** |
| --- | --- | --- | --- | --- |
| Library | 1b | Female | CI1 | Chronic HCV (CI) |
| Library | 1b | Female | CI2 |  |
| Library | 1b | Female | CI3 |  |
| Library, General repertoire | 1b | Female | CI4 |  |
| Library | 1b | Female | CI5 |  |
| Library, General repertoire | 1b | Female | CI6 |  |
| Library, General repertoire | 1b | Female | CI7 |  |
| Library | 1b | Male | CI8 |  |
| Library | 6g | Male | CI9 |  |
| Library, General repertoire | 3a | Female | CI10 |  |
| Library, General repertoire | 1b | Male | CI11 |  |
| Library | 1b | Female | CI12 |  |
| Library, General repertoire | 3a | Male | CI13 |  |
| Library | 3a | Male | CI14 |  |
| Library, General repertoire | 1b | Male | CI15 |  |
| Library, General repertoire | 1a | Male | CI16 |  |
| Library, General repertoire | 1b | Female | CI17 |  |
| Library | 1b | male | CI18 |  |
| Library | 1b | male | CI19 |  |
| Library, General repertoire | 3a | Female | CI20 |  |
| Library, General repertoire | 1b | Male | CI21 |  |
| Library, General repertoire | 1b | Male | CI22 |  |
| Library, General repertoire | 1b | Male | CI23 |  |
| General repertoire | 1b | Female | CI25 |  |
| General repertoire | 1b | Male | CI26 |  |
| Specific repertoire | 1b | Female | CI51 |  |
| Specific repertoire | 1a | Male | CI55 |  |
| Specific repertoire | 1b | Male | CI56 |  |
| Specific repertoire | 1b | Female | CI57 |  |
| Specific repertoire | 1b | Male | CI58 |  |
| Specific repertoire | 1b | Female | CI59 |  |
| Specific repertoire | 1b | Male | CI60 |  |
| Specific repertoire | 1b | Female | CI61 |  |
| Specific repertoire | 1 | Male | CI65 |  |
| Specific repertoire | 1 | Male | CI66 |  |
| Library, General repertoire | N/A | Female | SC1 | Cleared HCV (SC) |
| Library, General repertoire | N/A | Female | SC2 |  |
| Library, General repertoire | N/A | Male | SC3 |  |
| Library | N/A | Male | SC4 |  |
| Library | N/A | Female | SC5 |  |
| Library | N/A | Male | SC6 |  |
| Library, General repertoire | N/A | Male | SC7 |  |
| Library, General repertoire | N/A | Male | SC8 |  |
| General repertoire | N/A | Female | SC9 |  |
| General repertoire | N/A | Male | SC10 |  |
| General repertoire | N/A | Female | SC11 |  |
| General repertoire | N/A | Female | SC12 |  |
| Library, General repertoire | N/A | Female | SC14 |  |
| General repertoire, Specific repertoire | N/A | Female | SC15 |  |
| Specific repertoire | N/A | Female | SC16 |  |
| Specific repertoire | N/A | Female | SC17 |  |
| Specific repertoire | N/A | Female | SC18 |  |
| Specific repertoire | N/A | Male | C1 | Control (C) |
| Specific repertoire | N/A | Male | C2 |  |
| Specific repertoire | N/A | Male | C3 |  |
| General repertoire | N/A | Female | C4 |  |
| General repertoire | N/A | Female | C5 |  |
| General repertoire | N/A | Male | C6 |  |
| General repertoire | N/A | Male | C7 |  |
| General repertoire | N/A | Female | C8 |  |
| General repertoire | N/A | Male | C9 |  |
| General repertoire | N/A | Male | C10 |  |
