# Supplementary material for: Antibody Repertoire Analysis of Hepatitis C Virus Infections Identifies Immune Signatures Associated With Spontaneous Clearance
Source: Front Immunol. 2018 Dec 21;9:3004. doi: 10.3389/fimmu.2018.03004 (PMC6308210; doi:10.3389/fimmu.2018.03004)
Supplement: Supplementary Table 2 — List of primers. [file Table_2.DOCX]

**Supplementary table 2. List of primers**

| **Primer’s name** | **Sequence** |
| --- | --- |
| pSHOOTER-sec-E2-1a-SE | GGGAAAGGTACCGTCCTCTCTCGTGATCGAGGGTAGGCCTGAATTCAGTACCATGGCCGAAACCCACGTCACCGG |
| pSHOOTER-sec-E2-1a-AS | GGG AAT GCG GCC GCC TCG GAC CTG TCC CTG |
| TAB-RI | CCATGATTACGCCAAGCTTGGGAGCC |
| CBD-As | GAATTCAACCTTCAAATTGCC |
| Hu-VH-NcoI-BACK1-1 | TTTAAGCCATGGCC CAG GTB CAG CTK GTR CAR TCT GG |
| Hu-VH-NcoI-BACK1-2 | TTTAAGCCATGGCC CAR ATG CAG CTG GTG CAG TCT GG |
| Hu-VH-NcoI-BACK1-3 | TTTAAGCCATGGCC GAR GTS CAG CTG GTR CAG TCT GG |
| Hu-VH-NcoI-BACK2-1 | TTTAAGCCATGGCC CAG ATC ACC TTG AAG GAG TCT GG |
| Hu-VH-NcoI-BACK2-2 | TTTAAGCCATGGCC CAG GTC ACC TTG AGG GAG TCT GG |
| Hu-VH-NcoI-BACK2-3 | TTTAAGCCATGGCC CAG GTC ACC TTG AAG GAG TCT GG |
| Hu-VH-NcoI-BACK3-1 | TTTAAGCCATGGCC GAR GTR CAR CTG GTG GAG TCY GG |
| Hu-VH-NcoI-BACK3-2 | TTTAAGCCATGGCC CAG GTG CAG CTG GTG GAG TCT GG |
| Hu-VH-NcoI-BACK3-3 | TTTAAGCCATGGCC GAG GTG CAG CTG TTG GAG TCT GG |
| Hu-VH-NcoI-BACK3-4 | TTTAAGCCATGGCC GAG GTG CAG CTG GTG GAG WCT G |
| Hu-VH-NcoI-BACK4-1 | TTTAAGCCATGGCC CAG GTG CAR CTG CAG GAG TCG GG |
| Hu-VH-NcoI-BACK4-2 | TTTAAGCCATGGCC CAG CTG CAG CTG CAG GAG TCS GG |
| Hu-VH-NcoI-BACK6-1 | TTTAAGCCATGGCC CAG GTA CAG CTG CAG CAG TCA GG |
| Hu-JH-FORF1-2-4-5 | TCCTGCTGAGCC TGA GGA GAC RGT GAC CAG GGT KCC |
| Hu-JH-FORF3 | TCCTGCTGAGCC TGA AGA GAC GGT GAC CAT TGT CCC |
| Hu-JH-FORF6 | TCCTGCTGAGCC TGA GGA GAC GGT GAC CGT GGT CCC |
| Hu-JH-FORF1-2-4-5L | CCACCACCACCGGATCCTCCTCCTCCTGC TGAGCC TGA GGA GAC RGT GAC CAG GGT KCC |
| Hu-JH-FORF3L | CCACCACCACCGGATCCTCCTCCTCCTGC TGAGCC TGA AGA GAC GGT GAC CAT TGT CCC |
| Hu-JH-FORF6L | CCACCACCACCGGATCCTCCTCCTCCTGC TGAGCC TGA GGA GAC GGT GAC CGT GGT CCC |
| Hu-VK-BACKF1S | GGC GGC GGC TCC RHC ATC YRG WTG ACC CAG TC |
| Hu-VK-BACKF2-4S | GGC GGC GGC TCC GAY RTY GTG ATG ACY CAG WC |
| Hu-VK-BACKF3S | GGC GGC GGC TCC GAA ATW GTR WTG ACR CAG TC |
| Hu-VK-BACKF5S | GGC GGC GGC TCC GAA ACG ACA CTC ACG CAG TC |
| Hu-VK-BACKF6S | GGC GGC GGC TCC GAW RTT GTG MTG ACW CAG TC |
| Hu-VK-Lin-BACKF1L | GGATCCGGTGGTGGTGGT TCC GGA GGC GGC GGC TCC GGC GGC GGC TCC RHC ATC YRG WTG ACC CAG TC |
| Hu-VK-Lin-BACKF2-4L | GGATCCGGTGGTGGTGGT TCC GGA GGC GGC GGC TCC GGC GGC GGC TCC GAY RTY GTG ATG ACY CAG WC |
| Hu-VK-Lin-BACKF3L | GGATCCGGTGGTGGTGGT TCC GGA GGC GGC GGC TCC GGC GGC GGC TCC GAA ATW GTR WTG ACR CAG TC |
| Hu-VK-Lin-BACKF5L | GGATCCGGTGGTGGTGGT TCC GGA GGC GGC GGC TCC GGC GGC GGC TCC GAA ACG ACA CTC ACG CAG TC |
| Hu-VK-L-BACKF6L | GGATCCGGTGGTGGTGGT TCC GGA GGC GGC GGC TCC GGC GGC GGC TCC GAW RTT GTG MTG ACW CAG TC |
| Hu-JK-NotI-FORF1-3-4 | ATATATGCGGCCGC TTT GAT HTC CAC YTT GGT CC |
| Hu-JK-NotI-FORF2 | ATATATGCGGCCGC TTT GAT CTC CAG CTT GGT CC |
| Hu-JK-NotI-FORF5 | ATATATGCGGCCGC TTT AAT CTC CAG TCG TGT CC |
| Hu-VL-BACKF1S | GGC GGC GGC TCC CAG TCT GTS BTG ACK CAG CC |
| Hu-VL-BACKF2S | GGC GGC GGC TCC CAG TCT GCC CTG ACT CAG CC |
| Hu-VL-BACKF3S | GGC GGC GGC TCC TCY TMT GWG CTG ACW CAG CC |
| Hu-VL-BACKF3DEGS | GGC GGC GGC TCC TCC TAT GAG CTG AYH CAG SWV C |
| Hu-VL-BACKF4-5 | GGC GGC GGC TCC CAG SYT GTG CTG ACT CAA YC |
| Hu-VL-BACKF6S | GGC GGC GGC TCC AAT TTT ATG CTG ACT CAG CC |
| Hu-VL-BACKF7-8S | GGC GGC GGC TCC CAG RCT GTG GTG ACY CAG G |
| Hu-VL-BACKF9-10S | GGC GGC GGC TCC CWG SCW GKG CTG ACT CAG CC |
| Hu-VL-BACKF1L | GGATCCGGTGGTGGTGGT TCC GGA GGC GGC GGC TCC GGC GGC GGC TCC CAG TCT GTS BTG ACK CAG CC |
| Hu-VL-BACKF2L | GGATCCGGTGGTGGTGGT TCC GGA GGC GGC GGC TCC GGC GGC GGC TCC CAG TCT GCC CTG ACT CAG CC |
| Hu-VL-BACKF3L | GGATCCGGTGGTGGTGGT TCC GGA GGC GGC GGC TCC GGC GGC GGC TCC TCY TMT GWG CTG ACW CAG CC |
| Hu-VL-BACKF3DEGL | GGATCCGGTGGTGGTGGT TCC GGA GGC GGC GGC TCC GGC GGC GGC TCC TCC TAT GAG CTG AYH CAG SWV C |
| Hu-VL-BACKF4-5L | GGATCCGGTGGTGGTGGT TCC GGA GGC GGC GGC TCC GGC GGC GGC TCC CAG SYT GTG CTG ACT CAA YC |
| Hu-VL-BACKF6L | GGATCCGGTGGTGGTGGT TCC GGA GGC GGC GGC TCC GGC GGC GGC TCC AAT TTT ATG CTG ACT CAG CC |
| Hu-VL-BACKF7-8L | GGATCCGGTGGTGGTGGT TCC GGA GGC GGC GGC TCC GGC GGC GGC TCC CAG RCT GTG GTG ACY CAG G |
| Hu-VL-BACKF9-10L | GGATCCGGTGGTGGTGGT TCC GGA GGC GGC GGC TCC GGC GGC GGC TCC CWG SCW GKG CTG ACT CAG CC |
| Hu-JL-NotI-FORF1-2-3 | ATATATGCGGCCGC TAG GAC GGT SAC CTT SGT CCC |
| Hu-JL-NotI-FORF4 | ATATATGCGGCCGC TAG GAC GAT CAG CTG GGT TCC |
| Hu-JL-NotI-FORF5 | ATATATGCGGCCGC TAG GAC GGT CAG CTC SGT CCC |
| Hu-JL-NotI-FORF6-7 | ATATATGCGGCCGC TAG GAC GGT CAS CTK GGT KCC |
