# Supplementary material for: Antibody Repertoire Analysis of Hepatitis C Virus Infections Identifies Immune Signatures Associated With Spontaneous Clearance
Source: Front Immunol. 2018 Dec 21;9:3004. doi: 10.3389/fimmu.2018.03004 (PMC6308210; doi:10.3389/fimmu.2018.03004)
Supplement: Supplementary Table 3 — Clones detected in HCV-specific B cell repertoire and enriched in CI. [file Table_3.DOCX]

| **Clone** | **C** | **CI** | **SC** |
| --- | --- | --- | --- |
| IGHV1-18*IGHJ6*23**270 | 0 | 3 | 0 |
| IGHV3-21*IGHJ4*14**236 | 1 | 3 | 0 |
| IGHV3-21*IGHJ4*14**333 | 0 | 3 | 0 |
| IGHV3-21*IGHJ6*17**240 | 1 | 3 | 1 |
| IGHV3-30*IGHJ4*15**571 | 1 | 3 | 0 |
| IGHV3-33*IGHJ4*11**135 | 0 | 4 | 0 |
| IGHV3-33*IGHJ4*13**237 | 1 | 3 | 0 |
| IGHV3-33*IGHJ4*14**196 | 0 | 4 | 1 |
| IGHV3-33*IGHJ4*14**592 | 0 | 3 | 0 |
| IGHV3-48*IGHJ4*12**885 | 0 | 3 | 1 |
| IGHV3-48*IGHJ4*14**181 | 2 | 4 | 1 |
| IGHV3-7*IGHJ4*12**275 | 0 | 3 | 0 |
| IGHV3-7*IGHJ6*17**30 | 0 | 3 | 0 |
| IGHV4-34*IGHJ6*15**3 | 0 | 3 | 1 |
| IGHV4-34*IGHJ6*16**149 | 0 | 3 | 1 |

**Supplementary table 3. Clones detected in HCV-specific B cell repertoire and enriched in CI**

**Supplementary table 4. Clones detected in HCV-specific B cell repertoire and enriched in SC samples**

| **Clone** | **C** | **CI** | **SC** |
| --- | --- | --- | --- |
| IGHV1-18*IGHJ6*15**79 | 0 | 0 | 4 |
| IGHV1-18*IGHJ6*24**16 | 0 | 0 | 3 |
| IGHV1-2*IGHJ4*13**635 | 0 | 0 | 3 |
| IGHV1-8*IGHJ6*14**18 | 0 | 0 | 3 |
| IGHV3-23*IGHJ4*14**2188 | 0 | 0 | 4 |
| IGHV3-23*IGHJ4*15**138 | 2 | 1 | 4 |
| IGHV3-23*IGHJ4*15**1489 | 0 | 0 | 3 |
| IGHV3-33*IGHJ4*12**208 | 1 | 1 | 3 |
| IGHV3-33*IGHJ4*14**185 | 0 | 0 | 3 |
| IGHV3-33*IGHJ4*15**163 | 0 | 1 | 3 |
| IGHV3-33*IGHJ4*15**208 | 0 | 2 | 3 |
| IGHV3-33*IGHJ6*17**24 | 0 | 0 | 3 |
| IGHV3-48*IGHJ4*11**104 | 0 | 0 | 4 |
| IGHV3-9*IGHJ6*18**55 | 0 | 1 | 3 |
| IGHV4-39*IGHJ5*15**201 | 0 | 2 | 3 |
| IGHV4-59*IGHJ4*11**184 | 2 | 0 | 3 |
| IGHV6-1*IGHJ6*17**20 | 0 | 0 | 5 |
